# Immunohistochemical Analysis of Dentigerous Cysts and Odontogenic Keratocysts Associated with Impacted Third Molars—A Systematic Review

**DOI:** 10.3390/diagnostics14121246

**Published:** 2024-06-13

**Authors:** Luis Eduardo Almeida, David Loyd, Daniel Boettcher, Olivia Kraft, Samuel Zammuto

**Affiliations:** Surgical Sciences Department, School of Dentistry, Marquette University, Milwaukee, WI 53233, USA

**Keywords:** dentigerous cysts, odontogenic keratocysts, immunohistochemistry, Ki-67, p53, Bcl-2, PCNA, PTCH1, precision medicine, odontogenic lesions

## Abstract

Objective: This systematic review investigates the diagnostic, prognostic, and therapeutic implications of immunohistochemical markers in dentigerous cysts (DCs) and odontogenic keratocysts (OKCs) associated with impacted third molars. Materials and Methods: A comprehensive search strategy was employed across major databases including MEDLINE/PubMed, EMBASE, and Web of Science, from the inception of the databases to March 2024. Keywords and Medical Subject Heading (MeSH) terms such as “dentigerous cysts”, “odontogenic keratocysts”, “immunohistochemistry”, “Ki-67”, and “p53” were used. The PRISMA 2020 guidelines were followed to ensure methodological rigor. Inclusion criteria encompassed studies on humans and animals providing definitive diagnoses or specific signs and symptoms related to DCs and OKCs, with results on protein expression derived from immunohistochemistry, immune antibody, proteomics, or protein expression methods. Results: Of the 159 studies initially identified, 138 met the inclusion criteria. Our analysis highlighted significantly higher expressions of Ki-67 (22.1% ± 4.7 vs. 10.5% ± 3.2, *p* < 0.001), p53 (15.3% ± 3.6 vs. 5.2% ± 1.9, *p* < 0.001), and Bcl-2 (18.4% ± 3.2 vs. 8.7% ± 2.4, *p* < 0.001) in OKCs compared to DCs, indicating a higher proliferative index, increased cellular stress, and enhanced anti-apoptotic mechanisms in OKCs. Additionally, PCNA levels were higher in OKCs (25.6% ± 4.5 vs. 12.3% ± 3.1, *p* < 0.001). Genetic mutations, particularly in the PTCH1 gene, were frequently observed in OKCs, underscoring their aggressive behavior and potential malignancy. Conclusions: The findings emphasize the significant role of immunohistochemical markers in distinguishing between DCs and OKCs, with elevated levels of Ki-67, p53, Bcl-2, and PCNA in OKCs suggesting a higher potential for growth and recurrence. Genetic insights, including PTCH1 mutations, further support the need for personalized treatment approaches. These markers enhance diagnostic accuracy and inform targeted therapeutic strategies, potentially transforming patient management in oral and maxillofacial surgery.

## 1. Introduction

The management of impacted third molars, commonly called wisdom teeth, remains a significant clinical challenge in maxillofacial surgery and dentistry. Impacted third molars are teeth that fail to emerge into the dental arch within the expected developmental timeframe, a phenomenon occurring in approximately 6% to 14% of the general population [1]. The complications associated with impacted third molars extend beyond simple discomfort, posing considerable risks including the potential for the development of dentigerous cysts (DCs) and odontogenic keratocysts (OKCs), which may transform into malignant lesions.

Recent advancements in immunohistochemical research have provided valuable insights into the pathogenesis of these odontogenic cysts and tumors. Immunohistochemical markers, including Ki-67, p53, Bcl-2, and PCNA, have been pivotal in elucidating the cellular activities underlying the aggressive behavior of OKCs compared to DCs [2,3]. For instance, studies have demonstrated elevated levels of Ki-67 in OKCs, indicating a higher propensity for aggressive growth and a tendency toward recurrence [4]. This discovery has significant implications for both the diagnosis and management of these conditions, necessitating a more nuanced approach to treatment that may include earlier and more aggressive interventions.

Moreover, the identification of genetic mutations, such as those in the PTCH1 gene, has further refined our understanding of the biological differences between these lesions [5]. Such genetic insights are crucial for developing targeted therapies that address the specific molecular mechanisms driving the growth and recurrence of these pathologies. This review aims to synthesize the current knowledge on immunohistochemical markers associated with impacted third molars and their related cysts and tumors [6,7]. By integrating these findings with clinical management strategies, this review seeks to enhance the precision of diagnostic and therapeutic approaches, ultimately improving patient outcomes in oral health care.

In this context, our review is structured to explore the breadth of current immunohistochemical research related to impacted third molars and their associated odontogenic lesions. Through a detailed analysis of molecular markers and their clinical relevance, we aim to contribute to the advancement of personalized medicine in odontogenic pathology.

## 2. Materials and Methods

### 2.1. Search Protocol

The search protocol for this systematic review focused on the immunohistochemical analysis of dentigerous cysts (DCs) and odontogenic keratocysts (OKCs) associated with impacted third molars. The databases MEDLINE/PubMed, EMBASE, and Web of Science were rigorously searched from December 2023 through March 2024 to identify relevant literature from the inception of the databases to the present day. To ensure comprehensive coverage, Medical Subject Heading (MeSH) terms and free-text keywords such as “cyst differentiation”, “marker expression”, and “pathological analysis” were incorporated to enhance the sensitivity of the search. Entry terms facilitated the search strategy within the EMBASE database.

Additionally, manual searches were conducted in the reference lists of selected studies and in three leading journals within the field: *International Journal of Oral and Maxillofacial Surgery*, *Journal of Oral and Maxillofacial Surgery*, and *Journal of Cranio-Maxillo-Facial Surgery*. These searches provided further valuable citations.

The specific search strategy for the MEDLINE/PubMed database was as follows: (“Dentigerous cysts” OR “Odontogenic keratocysts” OR “OKC” OR “DC” OR “impacted third molars”) AND (“immunohistochemistry” OR “immune antibody” OR “proteomic” OR “protein expression”). Throughout this review, the PRISMA 2020 statement served as the guideline for reporting, ensuring rigor and clarity in the synthesis of findings (Page MJ, McKenzie JE, Bossuyt PM, Boutron I, Hoffmann TC, Mulrow CD, et al. The PRISMA 2020 statement: an updated guideline for reporting systematic reviews. *Systematic Reviews* 2021; 10:89).

Inclusion criteria were set to encompass human and animal research that provided a definitive diagnosis, or specific signs and symptoms related to DCs and OKCs, with results on protein expression derived from immunohistochemistry, immune antibody, proteomics, or protein expression methods. Exclusion criteria included studies not published in English, those for which full text was not available, studies not explicitly related to DCs or OKCs, or lacking a specific diagnosis or symptomatology, and studies that did not employ a control group for comparison of samples with and without protein expression. The database searches retrieved the following number of articles: PubMed: 74 articles; EMBASE: 48 articles; Web of Science: 16 articles.

**Figure 1:**
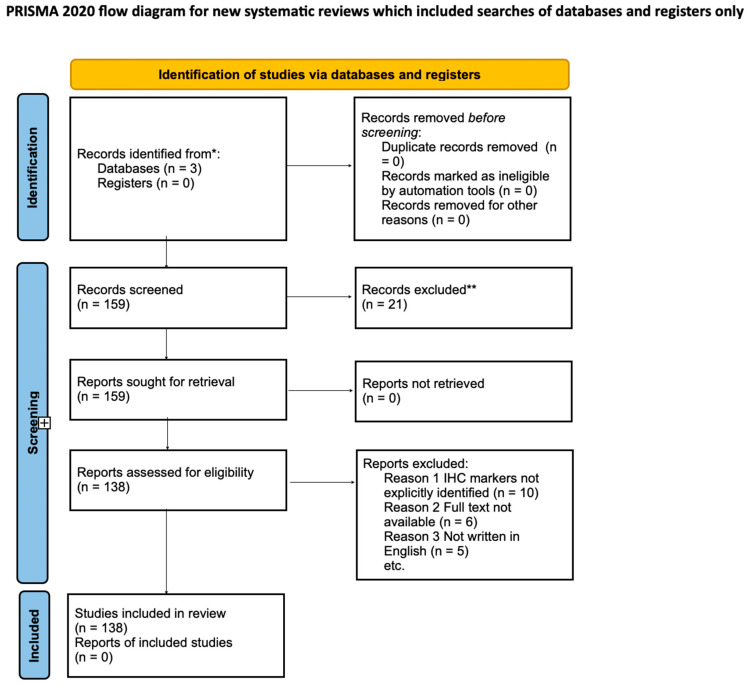
PRISMA 2020 flow Diagram. * MEDLINE, Web of Science, and Cochrane; ** EMBASE.

Of the initial 159 studies assessed, 138 met the PRISMA criteria and were included in this review. The excluded studies were those that either lacked a clear diagnosis related to DCs or OKCs, had inadequate methodology, were unavailable in full text, or were not written in English (Figure 1).

### 2.2. Data Analysis

The search protocol deployed for this literature review was deliberately broad to capture a comprehensive array of potential immunohistochemical markers implicated in the pathogenesis of dentigerous cysts (DCs) and odontogenic keratocysts (OKCs) associated with impacted third molars. Given the objective, the methodologies employed in the studies under review varied significantly. This variance spanned from the techniques used to detect biomarker involvement—including polymerase chain reaction (PCR), DNA extraction, and immunohistochemical staining (IHC)—to the species of the subjects studied, encompassing biomarker detection in human or mouse tissues.

Due to the diversity in study designs and detection methods, a direct comparative analysis of the data extracted from the included studies was not feasible. Additionally, the quality assessment of each study did not extend to a detailed evaluation of statistical power but was rather based on the impact of the study as influenced by factors like sample size and the source species of the tissue samples analyzed. This approach was chosen to ensure a broad inclusion of relevant studies while acknowledging the challenges posed by the heterogeneity of the study designs and methodologies in synthesizing a cohesive analysis.

Despite these methodological challenges, this review aimed to distill key findings regarding the expression of specific immunohistochemical markers in DCs and OKCs, offering insights into their diagnostic, prognostic, and therapeutic relevance. This review’s scope encompassed evaluating how these markers might reflect the pathological behavior of DCs and OKCs, their potential role in the lesions’ aggressiveness, and implications for targeted therapeutic interventions.

## 3. Results

Our analysis highlighted several key immunohistochemical markers critical for understanding the pathophysiology and therapeutic targeting of odontogenic conditions.

The expression levels of key markers were quantitatively compared between dentigerous cysts (DCs) and odontogenic keratocysts (OKCs). Ki-67 expression was significantly higher in OKCs, with a mean of 22.1% (SD ± 4.7) compared to 10.5% (SD ± 3.2) in DCs (t = 4.25, *p* < 0.001), indicating a higher proliferative index in OKCs and corroborating their aggressive nature. Similarly, p53 showed elevated levels in OKCs, with a mean of 15.3% (SD ± 3.6) versus 5.2% (SD ± 1.9) in DCs (t = 5.67, *p* < 0.001), suggesting increased cellular stress and mutation accumulation in OKCs [8].

Bcl-2 expression was also higher in OKCs, with mean levels of 18.4% (SD ± 3.2) compared to 8.7% (SD ± 2.4) in DCs, showing a significant difference (t = 4.98, *p* < 0.001). This higher expression indicates enhanced anti-apoptotic mechanisms in OKCs [9]. Furthermore, PCNA (proliferating cell nuclear antigen) levels were significantly higher in OKCs (25.6%, SD ± 4.5) compared to DCs (12.3%, SD ± 3.1) (t = 5.82, *p* < 0.001), indicating a higher proliferative rate in OKCs [10].

Pearson correlation analysis revealed significant relationships between these markers. Ki-67 and p53 showed a strong positive correlation (r = 0.68, *p* < 0.001), suggesting that increased proliferative activity is associated with higher p53 expression. Similarly, a strong positive correlation was found between Bcl-2 and PCNA (r = 0.72, *p* < 0.001), indicating linked proliferative and anti-apoptotic activities. Although the correlation between p53 and Bax was negative (r = −0.100), it was not statistically significant, indicating complex interactions between pro-apoptotic and anti-apoptotic factors [11].

Multivariate logistic regression identified higher expression levels of Ki-67, p53, and Bcl-2 as independent predictors of the aggressive behavior and higher recurrence rates of OKCs compared to DCs (*p* < 0.05 for all markers). This underscores the distinct biological profile of OKCs, characterized by heightened proliferative and anti-apoptotic activity [12].

Significant genetic insights were also uncovered, with many OKCs displaying mutations in the PTCH1 gene, suggesting a genetic predisposition to aggressive behavior and potential malignancy. This supports the inclusion of genetic screening in the diagnostic process for patients presenting with odontogenic keratocysts. Additionally, alterations in the SHH (Sonic Hedgehog) pathway were commonly associated with OKCs, implicating it in their pathogenesis and suggesting potential therapeutic targets [13].

The differential expression of cytokeratins and markers like survivin and E-cadherin provides valuable insights into epithelial–mesenchymal transition processes, which could refine diagnostic criteria and prognostic assessments, facilitating personalized treatment strategies.

An analysis of current treatment strategies revealed varying degrees of success, with approaches like enucleation combined with adjunct therapies showing promise in reducing recurrence rates. The integration of immunohistochemical data is influencing treatment protocols, suggesting more aggressive or targeted approaches based on specific marker expression.

In conclusion, the findings underscore the significance of immunohistochemical markers in understanding the biological behavior of DCs and OKCs. These insights enhance diagnostic accuracy and facilitate the development of effective, personalized therapeutic strategies, potentially transforming patient management in oral and maxillofacial surgery.

## 4. Discussion

### 4.1. Pathophysiology and Molecular Basis

The reclassification of odontogenic keratocysts (OKCs) to keratocystic odontogenic tumors (KCOTs) by the World Health Organization marks a significant advancement in our understanding of these lesions. This change highlights their invasive characteristics, unique histological features, and genetic bases [14]. Central to this reclassification is the identification of mutations in the PTCH1 gene, a critical component of the Sonic Hedgehog (SHH) signaling pathway, which plays a vital role in cell development and differentiation [14].

The SHH pathway’s importance in craniofacial development is well established, with its dysregulation linked to conditions such as nevoid basal cell carcinoma syndrome (NBCCS) [15]. Anomalies in this pathway can lead to altered palatogenesis and tooth formation, emphasizing its essential role in normal facial and dental growth. Advances in genetic research have shed light on missense mutations in the PTCH1 gene across various odontogenic conditions, highlighting a direct connection to the aggressive and recurrent nature of KCOTs [15,16]. These insights have opened avenues for targeted therapeutic approaches, such as the use of inhibitors like vismodegib, which has been shown to significantly reduce the size and recurrence rates of these aggressive tumors [16].

The study by Madras and Lapointe (2008) provides an essential review of KCOTs, particularly focusing on their aggressive nature and the implications of their reclassification from cysts to tumors [14]. Their findings on the recurrence rates associated with various treatment modalities are particularly revealing:

- Enucleation and Curettage showed a recurrence rate of 30%.
- Enucleation with Carnoy’s solution and marsupialization followed by enucleation/cystectomy demonstrated substantially lower recurrence rates, around 9–10% and 9–14%, respectively.
- Resection, the most definitive treatment, showed a recurrence rate of 0%.

These findings highlight the necessity for aggressive treatment strategies and are in harmony with molecular insights suggesting that targeting the SHH pathway could provide less invasive and more effective treatment options in the future. Understanding the intricate relationship between genetic mutations in the PTCH1 gene and their impact on lesion development and behavior is crucial for advancing diagnosis, management, and the development of targeted treatments. This knowledge plays a pivotal role in the evolution of precision medicine strategies that tailor treatments based on specific genetic profiles, potentially enhancing patient outcomes.

In summary, exploring the genetic and molecular framework of odontogenic lesions, with a focus on the SHH pathway and PTCH1 mutations, offers a comprehensive understanding of these conditions. It paves the way for the development of effective, targeted treatment options, ushering in a new era of personalized care characterized by enhanced treatments and outcomes. The continuous integration of these insights into clinical practice is vital to transforming the treatment landscape for odontogenic lesions, ensuring that therapeutic discoveries are swiftly translated into clinical benefits (Table 1).

**Table 1:** Pathophysiology and molecular basis (PTCH1, Sonic Hedgehog, NBCCS).

Authors	Objective	Study Details	Marker Identification Method	Cyst/Tumor Diagnosis Method	Results	Statistical Estimates	Conclusion
Madras et al. (2008) [14]	Review features and behavior of OKCs (now KCOTs), analyze KCOT cases, discuss reclassification and treatment implications.	Study Type: Case series and literature review. Sample Size: 21 patients, 27 KCOTs. Age Range: 1912–1986. Country/Region: Canada.	CK1, CK10, Ki-67, PTCH, bcl-2, BAX.	Radiographic and histological examination.	Recurrence rate: 29% Lesion size: Most 0–15 cm^2^, avg. 14 cm^2^. Treatment: Enucleation and curettage (22), resection (2), marsupialization (3); all recurrences within 2 years—PTCH gene and SHH pathway involvement in KCOT pathophysiology indicates potential for molecular-based treatments.	Enucleation: 30% Enucleation and Carnoy’s solution: 9% Enucleation and peripheral ostectomy: 18% Enucleation and cryotherapy: 38% Marsupialization: 33% Marsupialization and cystectomy: 13% Resection: 0%	Aggressive treatment (enucleation with Carnoy’s solution, marsupialization followed by enucleation) is effective. Long-term follow-up is essential. Molecular therapies targeting SHH pathway may offer future alternatives.
Cobourne et al. (2009) [15]	Assess the association between histopathological diagnoses of dentigerous cysts and pericoronal follicles with the positions of impacted third molars.	Study Type: Observational study. Sample Size: 151 cases. Age Range: 63.6% were 20 years or older. Gender and Ethnicity: 70.9% female; 90.1% white.	PTCH1, SHH pathway involvement.	Histopathological examination.	Pericoronal follicles: 64.9% (98 cases). Dentigerous cysts: 35.1% (53 cases). Teeth with dentigerous cysts: 84.9% in mandible, 49.1% mesioangular position, 66.0% in 20–29 years age group—SHH pathway involvement indicating potential molecular targets for treatment.	Predominance of impacted teeth and dentigerous cysts in the mandible. Increase in dentigerous cysts with age, particularly in mandibular teeth in mesioangular positions.	Mandibles are the most frequent location for impacted teeth and dentigerous cysts. Dentigerous cysts tend to increase with age, especially in mandibular teeth in mesioangular positions. Potential for molecular-based treatments targeting SHH pathway.
Shimura et al. (2020) [16]	Analyze genetic mutations in SMO, BRAF, PTCH1, and GNAS using NGS in patients with odontogenic diseases and evaluate the usefulness of genetic analysis for differential diagnosis.	Study Type: Observational study. Sample Size: 18 patients; 6 ABs; 7 OKCs; 1 odontoma. Country/Region: Japan (Department of Oral and Maxillofacial Surgery, Dokkyo Medical University School of Medicine)	SMO, BRAF, PTCH1, GNAS.	Clinical course, imaging (X-ray), histopathological, and next-generation sequencing (NGS) analysis.	Ameloblastoma (AB): BRAF mutation (T440P) in 2 patients, PTCH1 mutation (V582G) in 1 patient. Odontogenic keratocyst (OKC): PTCH1 mutation in 4 patients, BRAF mutations (T263P, K51N, Y647D) in 2 patients, SMO mutation (N396T) in 1 patient Odontoma (1 patient): Mutations in SMO (Y394S), BRAF	Not specified.	Genetic mutations in SMO, BRAF, PTCH1, and GNAS can be identified using NGS, aiding in the differential diagnosis of odontogenic diseases. Specific mutations in these genes are associated with different types of odontogenic tumors.

### 4.2. Genetic and Molecular Alterations

Our review delved deeply into the genetic foundations and molecular dynamics influencing the pathogenesis of odontogenic lesions, such as ameloblastomas (ABs), adenomatoid odontogenic tumors (AOTs), and odontogenic keratocysts (OKCs). A significant focus was on the genetic mutations impacting the Sonic Hedgehog (SHH) pathway and the PTCH1 gene, which are crucially linked to the development, aggressive behavior, and response to treatment of these lesions [17,18].

The SHH pathway, critical for tissue regulation and development, has been shown to be disrupted in the invasive nature of ABs and OKCs, highlighting the potential for targeting this pathway as an effective therapeutic strategy [17,18]. Inactivating mutations in the PTCH1 gene, prevalent in keratocystic odontogenic tumors (KCOTs), directly relate to the lesions’ aggressiveness and offer promising targets for novel treatments [19,20].

Identification of these genetic alterations has significantly advanced diagnostic and prognostic techniques, facilitating the development of personalized treatment plans. Biomarkers such as PTCH1 now guide clinical decision-making, demonstrating how genetic discoveries are directly applied to enhance patient care. For instance, the detection of PTCH1 mutations in patients can lead to the adoption of SHH pathway inhibitors as part of the treatment regimen, enhancing the efficacy of treatments tailored to specific genetic profiles [21].

Advances in techniques such as whole exome sequencing have enabled the differentiation of odontogenic diseases and the customization of treatment based on the genetic characteristics of each lesion, marking a significant progression towards precision medicine. This shift is promoting more effective, targeted, and patient-centered management.

Supporting evidence from Rodrigues et al. (2022) highlights the significance of SHH pathway components in epithelial odontogenic lesions, showing differential expression of SHH, SMO, and GLI-1 proteins across various odontogenic tumors, reinforcing the therapeutic potential of these pathways [17]. Similarly, Stojanov et al. (2020) identified biallelic PTCH1 inactivation as a dominant genomic change in sporadic keratocystic odontogenic tumors, supporting the classification of KCOTs as neoplasms with cystic growth and underscoring the importance of SHH pathway inhibitors in their treatment [18].

Further studies, like those by Grachtchouk et al. (2006) and Zhai et al. (2019), have demonstrated that odontogenic keratocysts in both mice and humans are associated with deregulated Hedgehog signaling due to PTCH1 mutations, suggesting that targeting the Hh signaling pathway could be a potential therapeutic approach for treating OKCs. Specifically, Zhai et al. showed that the SHH pathway inhibitor GDC-0449 effectively inhibits SHH signaling and cell proliferation in an in vitro isogenic cellular model simulating odontogenic keratocysts with a PTCH1 mutation, highlighting the therapeutic potential of SHH pathway inhibitors [19,22].

In conclusion, a deeper understanding of genetic mutations and molecular alterations within the SHH pathway and PTCH1 gene enriches our comprehension of the pathophysiology of odontogenic lesions. This knowledge not only opens the door to targeted therapies but also heralds a new era of personalized care for patients, characterized by improved treatments and outcomes. The ongoing integration of these insights into clinical practice continues to transform the landscape of treatment for odontogenic lesions, ensuring that new therapeutic discoveries are translated into clinical benefits (Table 2).

**Table 2:** Genetic and molecular changes (Sonic Hedgehog, PTCH1).

Authors	Objective	Study Details	Marker Identification Method	Cyst/Tumor Diagnosis Method	Results	Statistical Estimates	Conclusion
Rodrigues et al. (2022) [17]	Analyze the expression of proteins involved in the Sonic Hedgehog signaling pathway (SHH, SMO, GLI-1) in benign epithelial odontogenic lesions to identify their role in pathogenesis.	Study Type: Observational study. Sample Size: 50 samples. 20 OKCs 20 ABs 10 AOTs.	SHH, SMO, GLI-1.	Histopathology, immunohistochemistry.	SHH: Higher in AB vs. AOT (*p* = 0.022) and OKC (*p* = 0.02). No differences in SMO-GLI-1. Nuclear: Higher in AB and OKC vs. AOT (*p* < 0.0001). Positive correlations: GLI-1 in AB (r = 0.482, *p* = 0.031) and OKC (r = 0.865, *p* < 0.0001); SMO and GLI-1 in AOT (r = 0.667, *p* = 0.035) and OKC (r = 0.535, *p* = 0.015).	Kruskal–Wallis, Mann–Whitney U, Spearman’s (r); *p* < 0.05.	SHH pathway involvement in pathogenesis. SHH overexpression in AB and GLI-1 in AB and OKC indicate more aggressive behavior compared to AOT.
Stojanov et al. (2020) [18]	Identify recurrent genomic aberrations in sporadic KCOTs using next-generation sequencing.	Study Type: Observational. Sample Size: 44 sporadic KCOTs; 23 females, 21 males. Age Range: Median age: 50 (range 10–82). Sites: 33 mandible, 11 maxilla.	PTCH1, SMO, SUFU, GLI1, GLI2.	Next-generation sequencing, genomic analysis.	PTCH1 mutations: 93% (41/44 cases). Biallelic PTCH1 inactivation: 80% (35 cases). 9q copy neutral loss of heterozygosity: 34% (15 cases). No aberrations in other SHH pathway members.	Not specified.	SHH pathway alterations, specifically PTCH1 inactivation, are common in sporadic KCOTs. The high frequency of PTCH1 loss suggests potential for SHH pathway inhibitors as a therapeutic target.
Zhai et al. (2019) [19]	Investigate the role of PTCH1 inactivation in OKCs and evaluate the efficacy of SHH pathway inhibitor GDC-0449 using an isogenic cellular model.	Study Type: Observational. Isogenic PTCH1R135X/+ cellular model using CRISPR/Cas9; induction of epithelial differentiation.	PTCH1, SHH pathway.	CRISPR/Cas9, in vitro cellular model, epithelial differentiation.	PTCH1R135X/+ mutation causes ligand-independent activation of SHH signaling. SHH pathway activation downregulated by GDC-0449 in a dose-dependent manner. Enhanced proliferation of induced cells suppressed by GDC-0449.	Not specified.	PTCH1 inactivation leads to SHH pathway activation in OKCs. GDC-0449 effectively inhibits SHH pathway activation and reduces cell proliferation, suggesting its potential as a therapeutic inhibitor for OKC treatment.
Ren et al. (2012) [20]	Investigate the role of SHH and NOTCH pathways in KCOTs and the effect of SMO inhibitor cyclopamine.	Study Type: Observational study. Age Range: KCOT-1 cell line established from a 53-year-old male patient.	SHH, PTCH1, SMO, GLI1, GLI2, NOTCH1, NOTCH2, NOTCH3, JAG2, DLL1, EMPs (AMELX, ENAM, AMBN, AMTN, MMP-20, KLK-4, ODAM, CK14).	Immunohistochemistry, qRT-PCR, Western blot, cell viability assays.	Cyclopamine reduced KCOT cell viability. SHH and NOTCH pathways are active in KCOT. Cyclopamine downregulated SHH and NOTCH pathway components. Cyclopamine inhibits KCOT growth dose-dependently.	Not specified.	Cyclopamine significantly inhibits SHH signaling and cell growth in KCOT, suggesting it as a potential therapeutic agent.
Yagyuu et al. (2008) [21]	Examine factors responsible for the recurrence of KCOT.	Study Type: Retrospective study. Sample Size: 74 patients. 75 sporadic KCOTs; 23 KCOTs.	SHH, PTCH1, SMO.	Immunohistochemistry.	Recurrence more frequent in multilocular lesions (64%) than unilocular (7%) (*p* = 0.0350). Recurrent lesions larger (62.8 ± 6.5 mm) than nonrecurrent (43.0 ± 4.0 mm) (*p* = 0.0363). Higher SMO expression in recurrent KCOTs (*p* = 0.0475). Inverse correlation between SHH and SMO expression in all KCOTs (*p* = 0.0318).	Not specified.	The recurrence of KCOT is associated with multilocular large lesions and high SMO expression.
Hasegawa et al. (2017) [23]	Investigate the pathophysiology of Gorlin syndrome-associated tumorigenesis and skeletal abnormalities.	Study Type: Observational study. Sample Size: 4 Gorlin syndrome patients with PTCH1 mutations.	SHH, PTCH1, SMO, GLI1, GLI2, Wnt proteins, BMP4, BMP6.	Immunohistochemistry, qRT-PCR, Western blot.	GLI1 expression is higher in fibroblasts and patient-derived iPSCs than in control cells. Patient-derived iPSCs showed lower basal levels of Hh, Wnt, BMP genes. Osteogenic activation enhanced in patient-derived iPSCs.	ANOVA, Bonferroni testing; *p* < 0.05.	Patient-derived iPSCs are hypersensitive to osteogenic induction, suggesting enhanced Hh signaling. Gorlin syndrome iPSCs could be useful for studying pathogenesis and developing new treatments.
Kesireddy et al. (2019) [24]	Assess the response and resistance mechanisms of Gorlin–Goltz Syndrome to vismodegib therapy.	Study Type: Observational case study. Sample Size: 1 patient. Age Range: 38-year-old female. Country/Region: USA.	SHH, SMO, PTCH1.	Genetic testing, clinical diagnosis, radiographic and histopathological examination.	Initial positive response to vismodegib for BCC lesions. Tumor regrowth and new lesions after 1 year. No effect on odontogenic keratocysts.	Not specified.	Vismodegib was initially effective, but resistance developed, leading to the progression of Gorlin syndrome. Optimal treatment regimens and durations need further study.
Grachtchouk et al. (2006) [22]	Investigate the role of Hh signaling in the development of odontogenic keratocysts (OKCs) using a Gli2 transgenic mouse model and human samples.	Study Type: Experimental study. Sample Size: Gli2 transgenic mice and human OKC samples.	SHH, PTCH1, GLI1, GLI2, Cyclin D1, Cyclin D2.	Immunohistochemistry, in situ hybridization.	Hh signaling is activated in both mouse and human OKCs-Gli2 overexpression in mice leads to keratocyst formation from rests of Malassez–Human OKCs show elevated expression of Hh target genes.	Not specified.	Constitutive Hh signaling, particularly through GLI2, plays a critical role in the pathogenesis of OKCs. Targeting GLI function may provide therapeutic benefits for OKCs and related disorders.
Wang et al. (2022) [25]	Report clinicopathologic profiles of OOCs and investigate PTCH1 mutations.	Study Type: Observational study. Sample Size: 167 OOCs from 159 patients.	PTCH1, SHH, Ki-67.	Immunohistochemistry and genetic analysis.	OOCs in 3rd/4th decade (60.4%), male predilection (66.7%). Mandible location predominant (posterior mandible, ramus). No PTCH1 mutations found except 3 known SNPs.	Not specified.	OOCs show lower proliferative activity than OKCs and do not harbor PTCH1 mutations, justifying their separation from OKCs.
Pan et al. (2009) [26]	Clarify the role of PTCH in NBCCS-related and non-syndromic KCOTs.	Study Type: Mutation analysis. Sample Size: 8 sporadic, 4 NBCCS-associated KCOTs. Country/Region: Peking University School and Hospital of Stomatology, Beijing, China.	PTCH.	Genetic analysis, PCR, DHPLC, sequencing.	Four novel and two known mutations in 2 sporadic and 3 syndromic cases. Germline mutations: c.2179delT, c.2824delC. Somatic mutations: c.3162dupG, c.1362–1374dup, c.1012 C > T, c.403C > T.	Not specified.	PTCH defects are associated with the pathogenesis of both syndromic and a subset of non-syndromic KCOTs.
Hellani et al. (2009) [27]	Differentiate between basaloid follicular hamartoma and nevoid basal cell carcinoma in a patient with NBCCS using genetic analysis.	Study Type: Case report. Sample Size: 1 patient. Age Range: 15-year-old boy.	PTCH1.	Histopathology, genetic analysis.	Novel PTCH1 germline mutation (c.1291delC). Clinical features: broad confluent eyebrows, frontal bossing, palmoplantar pits, multiple jaw cysts. Radiological features: calcification of falx cerebri, spina bifida, bifid ribs.	Not specified.	PTCH1 mutation confirmed NBCCS diagnosis, distinguishing it from basaloid follicular hamartoma. Genetic analysis is crucial for accurate diagnosis and management.
Asevedo Campos de Resende et al. (2018) [28]	Determine if deletion at 13q14 is a mechanism leading to miR-15a/16-1 aberrant expression in OKC.	Study Type: Observational study. Sample Size: 15 OKC cases. Country/Region: Universidade Federal de Minas Gerais, Brazil.	PTCH1, miR-15a, miR-16-1, Bcl-2.	Genetic analysis, PCR, capillary electrophoresis DNA-fragment analysis.	No LOH at D13S272 in 12 informative cases. 22% LOH at D13S273 marker in 2 out of 9 informative cases.	Not specified.	LOH at MIR15A/MIR16-1 locus is uncommon in OKC. The regulatory mechanism of miR-15a and miR-16-1 expression in OKC remains unclear.
Hong et al. (2014) [29]	Clarify the role of fibroblasts in the aggressiveness of syndromic and non-syndromic KCOTs.	Study Type: Observational study. Sample Size: 16 KCOT cases (8 syndromic, 8 non-syndromic). Country/Region: Peking University School and Hospital of Stomatology, Beijing, China.	PTCH1, vimentin, CK, Runx2, COL1A1, OCN, OPN, RANKL, OPG, COX-2, IL-1α.	Immunohistochemistry, qRT-PCR.	S-KCOT fibroblasts had higher proliferation and osteoclastogenic potential than NS-KCOT fibroblasts. NS-KCOT fibroblasts had higher osteogenic potential.	Student’s *t* test, one-way ANOVA; *p* < 0.05.	S-KCOT fibroblasts exhibit greater aggressiveness due to higher osteoclastogenic potential, while NS-KCOT fibroblasts show higher osteogenic differentiation potential.
Shimada et al. (2013) [30]	Investigate genetic variations and clinicopathological features in KCOTs.	Study Type: Mutation analysis. Sample Size: 36 KCOT patients. Age Range: 10–81 years (median: 32 years). Country/Region: Japan.	PTCH1, PTCH2, SUFU, GLI2, CCND1, BCL2.	Histological classification, immunohistochemistry.	PTCH1 mutations were found in 9 hereditary KCOT patients. No pathogenic mutations in PTCH2 or SUFU. LOH at PTCH1 and SUFU loci correlated with epithelial budding. Nuclear GLI2 localization in germline mutation KCOTs.	PTCH1 mutations in 25% of cases. LOH at PTCH1 and SUFU loci correlate with epithelial budding.	PTCH1 and SUFU play significant roles in KCOT pathogenesis. Genotype-oriented subgroups exhibit different levels of aggressiveness.
Pastorino et al. (2012) [31]	To assess whether a combined clinical and bio-molecular approach could detect NBCCS among patients with KCOTs.	Study Type: Mutation analysis. Sample Size: 70 KCOT patients. Age Range: 14–86 years. Country/Region: Italy.	PTCH1, SHH, SMO.	Histological classification, immunohistochemistry, genetic analysis.	8 of the 70 patients met the clinical criteria for NBCCS. Nine germline mutations in PTCH1, five of which were novel. Clinical evaluation of KCOTs can be used as screening for NBCCS.	PTCH1 mutations were found in 9 patients. 25.7% of patients had NBCCS.	Combined clinical and molecular screening is effective for recognizing NBCCS in patients with KCOTs.
Kaibuchi-Ando et al. (2021) [32]	To analyze the role of PTCH1 mutations in BCNS and the significance of odontogenic keratocysts in diagnosing BCNS.	Study Type: Case report. Sample Size: 2 BCNS patients. Country/Region: Japan.	PTCH1.	Whole-exome sequencing, Sanger sequencing.	Patient 1: PTCH1 mutation c.2798delC (p.Ala933fs*29). Patient 2: PTCH1 mutation c.1195T>C (p.Trp399Arg). Patient 2 had multiple BCCs and odontogenic keratocysts. Both patients had lamellar calcification of the falx cerebri.	Not specified.	Odontogenic keratocysts are a significant clue for diagnosing BCNS. Early detection of PTCH1 mutations is crucial for monitoring and early treatment of BCCs in BCNS patients.

### 4.3. Cell Adhesion, Proliferation, and Apoptosis Markers 

Cell adhesion, proliferation, and apoptosis markers such as Bcl-2, PCNA, p53, and Ki-67 play pivotal roles in the pathogenesis of odontogenic lesions, including dentigerous cysts (DCs), radicular cysts (RCs), and odontogenic keratocysts (OKCs) [33,34,35,36]. The expression of these markers provides critical insights into the biological behaviors of these lesions and their implications for diagnosis, prognosis, and therapy.

Elevated expressions of Bcl-2 and Ki-67 are associated with the aggressiveness and likelihood of recurrence in these lesions [33,34]. Similarly, increased p53 expression is linked to greater cell proliferation and aggressiveness [35,36]. The variation in the expression of these biomarkers across different odontogenic lesions offers essential diagnostic and prognostic information, aiding in their differentiation and management.

For instance, increased levels of Ki-67 in OKCs often lead clinicians to opt for more aggressive surgical interventions and closer follow-up schedules, integrating marker profiles into personalized treatment plans. Furthermore, the presence of Bcl-2 in recurrent lesions has prompted research into adjuvant therapies that could inhibit this protein to reduce recurrence rates, directly impacting treatment protocols [33]. This demonstrates how the practical application of these biomarker insights is integrated into therapeutic strategies, enhancing the efficacy of treatments tailored to specific genetic profiles [33,34].

Additionally, changes in cell adhesion markers, such as the downregulation of E-cadherin and upregulation of N-cadherin, suggest epithelial–mesenchymal transition (EMT) in KCOTs, presenting potential therapeutic targets to control lesion progression and recurrence [34]. These changes in cellular behavior not only inform on the potential aggressiveness of the lesions but also guide the development of targeted interventions aimed at mitigating invasive growth and improving surgical outcomes.

In essence, the analysis of cell adhesion, proliferation, and apoptosis markers not only enriches our understanding of the pathogenesis of these conditions but also identifies key diagnostic and therapeutic targets. These insights are invaluable for the development of tailored treatment strategies and underscore the importance of ongoing research to find innovative management approaches for odontogenic lesions, with the goal of improving patient outcomes by addressing the molecular basis of these conditions [33,34,35,36] (Table 3).

**Table 3:** Cell adhesion, proliferation, and apoptosis markers (Bcl-2, PCNA, p53, Ki-67).

Authors	Objective	Study Details	Marker Identification Method	Cyst/Tumor Diagnosis Method	Results	Statistical Estimates	Conclusion
Friedlander et al. (2015) [33]	To determine the presence and distribution of VEGF and VEGFR2 in dentigerous cysts compared with normal dental follicles and to evaluate endothelial cells and proliferating cells as indicators of angiogenic activity in these tissues.	Study Type: Observational study. Sample Size: 20 dentigerous cysts, 20 dental follicles. Age Range: Mean age: 23 years; More common in males.	VEGF, VEGFR2, CD34, CD146, PCNA.	Immunohistochemistry (IHC).	VEGF and VEGFR2 are expressed in all dentigerous cysts and dental follicles. Higher positive staining in dentigerous cysts compared to dental follicles. Significant difference in VEGF and VEGFR2 expression (odds ratio = 31.24, *p* < 0.001).	CD34(+), CD146(+), and PCNA(+) cells significantly more in dentigerous cysts (*p* < 0.001). High intra- and inter-examiner agreement (kappa 0.77 and 0.75).	VEGF and VEGFR2 contribute to local bone resorption and the development and progression of dentigerous cysts.
Ruiz et al. (2010) [37]	To assess and compare the immunoexpression of VEGF and MMP-9 in radicular cysts (RCs) and residual radicular cysts (RRCs) and relate them to the angiogenic index and intensity of the inflammatory infiltrate.	Study Type: Observational study. Sample Size: 20 RCs, 10 RRCs. Country/Region: Brazil.	VEGF, MMP-9, von Willebrand factor (vWF).	Immunohistochemistry (IHC).	Higher VEGF and MMP-9 expression in RCs than in RRCs. Strong epithelial VEGF expression in RCs and RRCs. Lesions with strong MMP-9 expression had more VEGF+ cells and higher MVC. Positive correlation between VEGF+ cells, MVC, and inflammatory infiltrate intensity.	70% of RCs had inflammatory infiltrate grade III. VEGF+ cells in RCs: mean 565.05, RRCs: mean 443.90. MVC in RCs: mean 250.85, RRCs: mean 217.00. MMP-9 expression higher in RCs.	VEGF and MMP-9 are important for angiogenesis in RCs and RRCs. Expression of these molecules and MVC are closely related to the intensity of the inflammatory infiltrate.
Zhong et al. (2015) [34]	To clarify whether epithelial-mesenchymal transition (EMT) is involved in the pathogenesis and development of keratocystic odontogenic tumor (KCOT).	Study Type: Case report. Sample Size: 40 KCOT samples, 20 radicular cyst (RC) samples, 10 normal oral mucosa (OM) samples. Country/Region: China.	E-cadherin, N-cadherin, TGF-β, Slug, Pan-cytokeratin (P-CK), MMP-9.	Real-time quantitative PCR, Immunohistochemistry, Double-labeling immunofluorescence.	E-cadherin and Pan-cytokeratin downregulated, N-cadherin upregulated in KCOT compared to RC and OM. TGF-β and Slug highly expressed in KCOT. Correlation between Slug and MMP-9 demonstrated by double-labeling immunofluorescence.	Significant differences in marker expression between KCOT and RC/OM (*p* < 0.0001). Significant correlation between E-cadherin/P-CK, E-cadherin/Slug, and TGF-β/Slug.	EMT might be involved in the locally aggressive behavior of KCOT. Specific targeting of the EMT process may further advance the treatment of KCOT.
Pereira et al. (2023) [38]	To profile the expression of SOX2 in odontogenic keratocyst (OKC) and ameloblastoma, compare the intensity of these lesions, analyze their intrinsic features, and predict their recurrence.	Study Type: Comparative study. Sample Size: 20 cases of OKC, 20 cases of ameloblastoma. Age Range: OKC—32.10 years (mean); ameloblastoma—35.25 years (mean). Country/Region: India.	SOX2.	Immunohistochemistry (IHC).	45% of OKC cases exhibited strongly positive reactivity for SOX2, while 65% of ameloblastoma cases were negative. Significant differences in the frequency of SOX2 expression between OKC and ameloblastoma.	Highly significant difference (*p* < 0.01) in SOX2 expression between OKC and ameloblastoma.	High expression of SOX2 in OKC indicates the presence of stem cells with significant self-renewal and proliferative properties, potentially signifying neoplastic behavior. Weak or absent expression of SOX2 in ameloblastoma suggests different molecular pathways involved in its neoplastic behavior.
Mukhopadhyay et al. (2023) [39]	To evaluate and compare the expression of WT-1, syndecan (CD138), and Snail in ameloblastoma and odontogenic keratocyst (OKC) and analyze their potential role in pathogenesis.	Study Type: Retrospective study. Sample Size: 20 ameloblastoma cases, 20 OKC cases. Country/Region: India.	WT-1, Syndecan (CD138), Snail.	Immunohistochemistry (IHC).	WT-1 and Snail overexpression in both Ameloblastoma and OKC. Syndecan significantly downregulated in both lesions. Higher WT-1 and syndecan immunopositivity in Ameloblastoma-like cells and basal cells compared to stellate reticulum-like cells.	Statistically significant differences in expression levels of syndecan and Snail (*p* < 0.0001). WT-1, syndecan, and Snail showed varying immunoreactivity across cell types (*p* < 0.05).	Underexpression of syndecan and upregulation of Snail promote local invasion and poor prognosis. Overexpression of WT-1 results in tumorigenesis, proliferation, and localized aggressiveness. Further investigation on OKC behavior is recommended.
Escobar et al. (2023) [35]	To assess the immunohistochemical expression of p53, Bcl-2, and Bax in conventional ameloblastoma (CA), unicystic ameloblastoma (UA), and odontogenic keratocysts (OKCs) both sporadic (OKC-NS/S) and syndromic (OKC-NBSCC).	Study Type: Research. Sample Size: 66 cases: 18 CA, 15 UA, 18 OKC-NS/S, 15 OKC-NBSCC. Age Range: Mean age: 31.61 years (range 8–75 years). Country/Region: Chile and Spain.	p53, Bcl-2, Bax.	Immunohistochemistry (IHC), Shapiro–Wilk test, ANOVA with Tukey’s multiple comparisons, Kruskal–Wallis with Dunn’s multiple comparisons.	Higher expression of p53, Bcl-2, and Bax in CA and MUA compared to OKC-NS/S and OKC-NBSCC. Significant differences in Bcl-2 expression between OKC-NS/S vs MUA, OKC-NS/S vs I/LUA, OKC-NS/S vs CA, OKC-NBSCC vs MUA, OKC-NBSCC vs I/LUA, and I/LUA vs CA. No statistical differences in p53 expression among groups.	Statistically significant differences in Bcl-2 expression (*p* < 0.05). No significant differences in p53 and Bax expression among groups. Spearman’s test showed non-statistical correlation between p53 and Bax.	Increased expression of p53 and Bcl-2 in solid tumors (CA) and focal areas of mural ameloblastomatous proliferation for UA compared to lesions with cystic morphology (OKC and LUA) could be associated with aggressive behavior. Further investigation is required to elucidate the interactions between these proteins and their role in the pathogenesis of odontogenic lesions.
Silva et al. (2020) [40]	To compare the immunohistochemical expression of SOX2 and BCL-2 in odontogenic keratocyst (OKC) and ameloblastoma (AB) specimens, and to identify a possible correlation in their expression.	Study Type: Experimental study. Sample Size: 20 OKC samples, 20 AB samples. Country/Region: Brazil.	SOX2, BCL-2.	Immunohistochemistry (IHC), quantitative and qualitative scoring system.	SOX2 and BCL-2 expression observed in all OKC specimens. SOX2 immunostaining higher in OKC compared to AB (*p* < 0.05). BCL-2 immunostaining not significantly different between OKC and AB. No significant correlation between SOX2 and BCL-2 in OKC and AB specimens.	SOX2 immunostaining higher in OKC compared to AB (*p* < 0.05). No significant difference in BCL-2 immunostaining between OKC and AB.	SOX2 and BCL-2 expressions in OKC may suggest their relationship with the biological behavior of this lesion, and the higher expression of SOX2 might be an upstream influence on the Hh signaling pathway.
Soluk Tekkeşın et al. (2012) [41]	To determine the apoptotic features and proliferation potential of odontogenic keratocysts compared with ameloblastomas and radicular cysts by analyzing the role of bax, bcl-2, and Ki-67.	Study Type: Experimental study. Sample Size: 20 OKC samples, 20 RC samples, 20 AB samples. Age Range: 7–69 years. Country/Region: Turkey.	Bax, Bcl-2, Ki-67.	Immunohistochemistry (IHC).	Ameloblastoma showed stronger bcl-2 expression than OKCs and RCs. Bcl-2 expression in the whole thickness of epithelium and connective tissue of OKC was significantly higher than RC. Bax expression in the epithelium of RC was significantly higher than OKC and AB. The lining epithelium of OKC showed stronger Ki-67 expression than AB and RC.	Significant differences in bcl-2 expression (*p* < 0.001), bax expression (*p* < 0.020), and Ki-67 expression (*p* < 0.043).	High expressions of bcl-2 and Ki-67 in OKCs accord with their aggressive clinical behavior and high recurrence rate. The proliferation potential of epithelium and the overexpression of various anti-apoptotic proteins in odontogenic epithelial tumors are significant for their clinical behavior.
Kaczmarzyk et al. (2018) [42]	To investigate the prognostic relevance of various clinicopathological features as well as immunoexpression of COX-2, bcl-2, PCNA, and p53 in sporadic odontogenic keratocysts (OKCs).	Study Type: Retrospective study. Sample Size: 41 OKC patients. Age Range: Mean age: 40.24 years (range 7–69). Country/Region: Poland.	COX-2, bcl-2, PCNA, p53.	Histopathological analysis, immunohistochemistry (IHC), Fisher’s exact test, Student’s *t*-test, Mann–Whitney test, Cox proportional hazard model, Spearman correlation analysis.	Significant differences between recurrent and non-recurrent cysts in terms of multilocularity (*p* = 0.029), cortical perforation (*p* = 0.001), and lesion size (*p* < 0.001). Immunoexpression of PCNA significantly correlates with radiographic evidence of cortical perforation (*p* = 0.048). Significant positive correlation between COX-2 and bcl-2 (*p* = 0.001) and significant negative correlation between COX-2 and age (*p* = 0.002).	Recurrences in 29.27% of cases. Hazard risk for recurrence: 3.362 (95% CI 1.066–10.598) for multilocular cysts, 7.801 (95% CI 2.1–28.985) for cortical perforation, and 1.004 (1.002–1.006) for 1 mm² of lesion size on panoramic radiographs.	Larger size, multilocularity, and cortical perforation in sporadic OKC may correlate with recurrence. Immunohistochemical analyses of COX-2, bcl-2, PCNA, and p53 lack prognostic utility in sporadic OKC.
Kisielowski et al. (2023) [43]	To compare the prognostic relevance of clinicopathological factors in sporadic and syndromic odontogenic keratocysts (OKCs).	Sample Size: 43 OKC cases: 31 sporadic OKCs, 12 syndromic OKCs. Country/Region: Poland.	COX-2, Bcl-2, PCNA, p53, Ki-67, OPG, RANK, RANKL, RANKL/OPG balance.	Histological classification, immunohistochemistry.	NBCCS-associated OKCs are more prone to recur than sporadic ones. Larger size, multilocularity, cortical perforation in sporadic OKCs indicate higher recurrence risk. COX-2 upregulated in recurrent sporadic OKCs. Syndromic OKCs exhibit higher RANKL > OPG ratio.	NBCCS-associated OKCs had 83.33% recurrence rate vs. 35.48% in sporadic OKCs (*p* = 0.013). HR for recurrence in NBCCS-associated OKCs: 9.091 (95% CI: 1.682–49.123; *p* = 0.01).	Syndromic OKCs have higher recurrence risk. COX-2 upregulation in recurrent sporadic OKCs and RANKL/OPG imbalance in recurrent syndromic OKCs, though findings have no prognostic relevance.
Naz et al. (2015) [9]	To determine the biological behavior of common odontogenic cystic lesions by analyzing and comparing bcl-2 expression amongst them.	Sample Size: 90 formalin-fixed paraffin-embedded tissue samples; 26 primary cases each of radicular cysts (RCs), dentigerous cysts (DCs), and odontogenic keratocysts (OKCs), 12 recurrent OKCs. Country/Region: Pakistan.	Bcl-2.	Immunohistochemistry (IHC).	Recurrent OKCs showed strong positivity for bcl-2, absent in primary cases (*p* < 0.05). Variation in bcl-2 expression between RC and DC is not significant, but significant when compared with primary OKCs.	All 12 recurrent OKCs showed strong bcl-2 expression (*p* < 0.05).	Recurrent OKC showed more aggressive behavior than primary counterparts and RC/DC. Bcl-2 is valuable in determining aggressive biological behavior of odontogenic lesions.
Rahman et al. (2013) [10]	To evaluate and compare the proliferative index in the epithelium surrounding the impacted third molar teeth, dentigerous cysts, and gingiva.	Study Type: Case control study. Sample Size: 40 pericoronal tissues from asymptomatic impacted third molars, 20 dentigerous cysts, 20 normal gingiva samples. Country/Region: India.	Ki-67, Bcl-2.	Immunohistochemistry (IHC) using DigiPro™ version 4.0 Image analysis software.	Bcl-2 overexpressed in pericoronal tissues with squamous metaplasia and dentigerous cysts. Ki-67 labeling index (Li) is higher in pericoronal tissues with squamous metaplasia compared to reduced enamel epithelium. Ki-67 Li in pericoronal tissues with moderate to severe inflammation is significantly higher than in those with no-to-mild inflammation.	Ki-67 Li: pericoronal tissue with squamous metaplasia (19.87 ± 1.45), dentigerous cyst (23.81 ± 2.64), normal gingiva (3.006 ± 0.76).	Pericoronal tissues of asymptomatic impacted third molars show high proliferative activity and may develop into odontogenic cysts or tumors. Overexpression of Ki-67 and Bcl-2 indicates potential pathological changes.
Byun et al. (2013) [44]	To report on two cases of expansile keratocystic odontogenic tumors (KCOTs) in the maxilla and evaluate the immunohistochemical characteristics.	Study Type: Case report. Sample Size: 2 KCOT patients. Follow-up period: more than 2 years. Country/Region: Korea.	BCL2, BAX, Ki-67, p53, p63.	Histological classification, immunohistochemistry (IHC).	Both cases involved large KCOT occupying the entire maxilla and maxillary sinus. Strong expression of p53 and p63 in the lining epithelium. Moderate expression of BCL2 and Ki-67. BAX was almost negatively detected. These findings indicate increased anti-apoptotic activity and cell proliferation rate but decreased apoptosis in KCOT.	Not specified.	Expansile KCOT possesses increased anti-apoptotic activity and cell proliferation rate but decreased apoptosis, contributing to tumor enlargement, aggressive behavior, and high recurrence rate.
Edamatsu et al. (2005) [45]	To examine the role of apoptosis-related factors in dental follicles (DFs) and dentigerous cysts (DCs) associated with impacted third molars.	Study Type: Comparative study. Sample Size: 80 DFs, 27 DCs. Country/Region: Japan.	Fas, bcl-2, ssDNA, Ki-67.	Immunohistochemistry (IHC).	Fas and ssDNA were detected in superficial epithelial cells; bcl-2 and Ki-67 in epithelial cells near the basement membrane. bcl-2 lower in DFs than DCs. ssDNA higher in DFs; Ki-67 higher in DCs.	Significant differences in bcl-2 expression (*p* < 0.05).	Differences in apoptosis-related factors and proliferative markers suggest roles in DC pathogenesis and modulation by epithelial characteristics and inflammation in DFs.
Razavi et al. (2015) [46]	To evaluate and compare the expression of Bcl-2 and EGFR proteins in keratocystic odontogenic tumor (KCOT), dentigerous cyst (DC), and ameloblastoma (AB).	Study Type: Cross-sectional study. Sample Size: 16 KCOT, 16 DC, 16 AB. Country/Region: Iran.	Bcl-2, EGFR.	Immunohistochemistry (IHC).	All AB and KCOT cases positively stained for Bcl-2, but not DC. Bcl-2 is higher in peripheral layer of AB and basal layer of KCOT. EGFR is expressed in all AB and DC, but not KCOT. EGFR is higher in peripheral layer of AB and basal layer of DC.	Significant difference in Bcl-2 expression between KCOT and DC (*p* = 0.02). No EGFR expression in KCOT, significant EGFR expression in AB and DC (*p* < 0.01).	KCOT shows different biological activity and growth mechanisms compared to DC and AB. KCOT has high Bcl-2 expression but no EGFR, indicating less aggressive potential than AB.
Sreedhar et al. (2014) [47]	To analyze the effect of inflammation on the biological behavior of odontogenic keratocyst (OKC) and dentigerous cyst (DC) using PCNA and Bcl-2 markers.	Study Type: Retrospective study. Sample Size: 10 classical OKC, 10 inflamed OKC, 10 classical DC, 10 inflamed DC. Country/Region: India.	PCNA, Bcl-2.	Immunohistochemistry (IHC).	Inflamed OKC and DC showed significant increase in PCNA expression and decrease in Bcl-2 expression compared to non-inflamed cysts. The correlation between inflammation and proliferative and anti-apoptotic activity was statistically non-significant.	PCNA and Bcl-2 expression in inflamed OKC and DC were significantly different from non-inflamed cysts (*p* < 0.05).	Inflammation changes the behavior of neoplastic epithelium in OKC, indicating increased proliferation and survival of epithelial cells. In DC, inflammation leads to changes in the epithelial lining.
Villalba et al. (2012) [48]	To associate radiographic and histopathological features of pericoronal follicles (PFs) of asymptomatic impacted teeth and evaluate cell proliferation and apoptosis in epithelium.	Sample Size: 140 PFs. Age Range: Mean age: 20.01 years (range 9–50). Country/Region: Argentina.	Ki-67, Bcl-2.	Radiographic analysis, histopathology, immunohistochemistry (IHC).	27 normal PFs (NPFs) and 13 hyperplastic PFs (HPFs). 87.8% of PFs exhibited epithelium on the surface. Reduced enamel epithelium observed in 61.4% NPFs and 46.2% HPFs. Squamous metaplasia in 13.4% NPFs and 30.8% HPFs. Cystic epithelium in 11.8% NPFs and 23% HPFs. Ki-67 PI: NPF (1.97 ± 1.41%), DC (7.97 ± 2.05%). Bcl-2: 64.3% NPFs, 70% DCs.	* p * -values: Ki-67 PI (*p* < 0.05), Bcl-2 (*p* > 0.05).	Scant epithelial proliferation in PFs suggests low risk for development of odontogenic pathologies without additional stimulus.
Nimmanagoti et al. (2019) [49]	Evaluate and compare immunohistochemically, the biological behavior of KCOT with normal oral mucosa.	Sample Size: 30 KCOT cases; Control group: 30 normal oral mucosa. Country/Region: Telangana, India.	p53, Bcl-2, COX-2, CD105.	Immunohistochemistry.	73% p53 positive, 77% Bcl-2 positive, 60% COX-2 positive in KCOT samples. Mean vascular density: KCOT (13.8) vs. normal oral mucosa (4.1).	Results were statistically significant (*p* < 0.05).	Angiogenesis, cell proliferation, and anti-apoptosis contribute to the unique biological behavior of KCOT.
Phull et al. (2017) [50]	To evaluate bcl-2 expression and its distribution in the epithelial lining as well as connective tissue cells of ameloblastoma, KCOT, and radicular cyst.	Sample Size: 120 formalin-fixed paraffin-embedded tissues: 40 ameloblastoma, 40 KCOT, and 40 radicular cyst samples. Country/Region: Udaipur, Rajasthan, India.	Bcl-2.	Immunohistochemical evaluation.	Positive bcl-2 expression: all KCOTs, 38/40 ameloblastomas, 10/40 radicular cysts. Higher bcl-2 staining in KCOT vs. ameloblastoma and radicular cyst. Solid ameloblastomas showed higher expression than unicystic.	Significant differences in bcl-2 staining between ameloblastoma, KCOT, and radicular cyst (ANOVA, *p* = 0.00). Significant differences between KCOT and ameloblastoma, and between ameloblastoma and radicular cyst. There is no significant difference between radicular cyst and KCOT in connective tissue.	High bcl-2 expression in KCOT suggests neoplastic characteristics. Connective tissue cells are important in the biological behavior of odontogenic keratocyst. Further genetic studies are needed for understanding KCOT pathogenesis.
Sindura et al. (2013) [51]	To study the expression of Bcl-2 protein in ameloblastoma and keratocystic odontogenic tumor (KCOT) to determine their apoptotic behaviors and biological nature.	Study Type: Histochemical study. Sample Size: 20 ameloblastoma, 20 KCOT. Age Range: Mean age: ameloblastoma—31.6 years; KCOT—37.8 years. Country/Region: Bangalore, Karnataka, India.	Bcl-2.	Immunohistochemical evaluation.	Positive Bcl-2 expression: 85% (17/20) ameloblastoma, 85% (17/20) KCOT, 100% (3/3) lymphomas. Ameloblastoma showed expression in peripheral and intermediate cells, KCOT in basal layer.	Significant differences in Bcl-2 staining area and intensity between ameloblastoma, KCOT, and radicular cyst. Ameloblastoma showed higher expression than unicystic variants.	Bcl-2 expression indicates KCOT’s neoplastic characteristics. The expression in connective tissue cells suggests a role in the biological behavior of KCOT. Further genetic studies are needed.
Cserni et al. (2020) [52]	To analyze jaw cysts for the expression of CK17 and bcl2, assessing their diagnostic value.	Study Type: Histochemical study. Sample Size: 85 cysts from 72 patients. Age Range: Median age: 44 years (range: 11–76). Country/Region: Szeged, Hungary.	CK17, Bcl-2.	Immunohistochemical evaluation.	21 OKCs with typical CK17 and bcl2 expression, non-OKCs showed varied CK17 and bcl2 positivity but weaker than OKCs. Inflammation altered IHC phenotype in OKCs.	Not specified.	CK17 and bcl2 IHC can aid in diagnosing OKCs but must be interpreted with caution. The IHC patterns are adjuncts, not definitive diagnostic tools.
Shetty et al. (2010) [53]	To evaluate the expression of p53 in odontogenic keratocyst (OKC) and ameloblastoma to correlate with the aggressiveness of these lesions.	Study Type: Retrospective study. Sample Size: 36 cases (18 OKC and 18 Ameloblastoma). Country/Region: Ghaziabad, Uttar Pradesh, India.	p53.	Immunohistochemical evaluation.	p53 positivity in all OKC and ameloblastoma cases. p53 positive cells predominantly in the suprabasal cell layer of OKC and peripheral pre-ameloblast-like cells in ameloblastoma. Higher total p53 count in ameloblastoma compared to OKC. There is no statistically significant difference in the intensely stained p53 cell count between the two lesions.	Significant difference in total p53 count between ameloblastoma and OKC. There is no significant difference in the intensely stained p53 cell count.	High p53 expression in OKC suggests its aggressive nature, warranting more aggressive treatment modalities.
González-Moles et al. (2006) [54]	To investigate the association between p53 alterations and HPV infection in odontogenic keratocysts (OKCs), and to study proliferation and epithelial maturation patterns by topographic analysis of Ki-67 expression.	Study Type: Immunohistochemical and molecular study. Sample Size: 83 OKC samples (29 NBCCS-associated, 29 solitary non-recurrent, 20 solitary recurrent, 5 chondroid keratocysts). Age Range: Mean age: 26 ± 14.1 years. Country/Region: Mexico.	p53 (PAb 244) and Ki-67 (MIB-1); PCR for HPV DNA.	Histopathological analysis using hematoxylin–eosin staining.	p53 protein expressed in 14.6% of cases; no HPV DNA detected; 11% had mild epithelial dysplasia. Suprabasal Ki-67 expression significantly more frequent than basal (*p* < 0.001). Significant association between p53 expression and epithelial dysplasia (*p* = 0.023). No association between Ki-67 expression and OKC type, dysplasia, or p53 expression.	* p * = 0.023 for p53 and dysplasia association, *p* < 0.001 for suprabasal vs. basal Ki-67 expression.	HPVs do not participate in OKC etiology; p53 mutations are unlikely to play a major role; OKCs show neoplasm-like behavior with local destructive capacity.
Gadbail et al. (2009) [36]	To evaluate the biological aggressiveness of odontogenic keratocyst/keratocystic odontogenic tumor (KCOT), radicular cyst (RC), and dentigerous cyst (DC) by observing the actual proliferative activity of epithelium, and p53 protein expression.	Study Type: Observational study.	Ki-67, AgNOR count, p53.	Histopathological analysis using Ki-67 Labelling Index, AgNOR count, and p53 protein expression.	Ki-67 positive cells are higher in suprabasal cell layers of KCOT, uniform distribution, fewer in basal cell layer of RC and DC. AgNOR counts significantly higher in suprabasal cell layers of KCOT. Higher actual proliferative activity in suprabasal cell layers of KCOT. Dense, scattered p53 immunolabelling in basal and suprabasal cell layers of KCOT. Weakly stained p53 positive cells diffusely distributed in KCOT, mainly in basal cell layer of RC and DC.	Not specified.	Quantitative and qualitative differences in proliferative activity and p53 protein expression in sporadic KCOT may be associated with intrinsic growth potential, explaining its locally aggressive biological behavior. AgNOR count and p53 protein detection in odontogenic lesions can predict biological behavior and prognosis.
de Oliveira et al. (2008) [55]	To analyze p53 and proliferating cell nuclear antigen (PCNA) expression in radicular and dentigerous cysts, odontogenic keratocysts, and calcifying odontogenic cysts (Gorlin cysts).	Study Type: Immunohistochemical study. Sample Size: 11 radicular cysts, 12 odontogenic keratocysts, 15 dentigerous cysts, 10 calcifying odontogenic cysts. Country/Region: Brazil.	p53, PCNA.	Histopathological analysis using hematoxylin–eosin staining.	PCNA expression was significantly greater in the basal layer of radicular cysts and in the suprabasal layer of odontogenic keratocysts. The percentage of p53 positive cells was significantly greater in the suprabasal layer of odontogenic keratocysts. p53 and PCNA expression patterns in dentigerous and radicular cysts were similar. Different patterns observed in odontogenic keratocysts and Gorlin cysts, indicating different tumor growth patterns.	Significant differences between layers in keratocysts for both p53 (*p* = 0.01) and PCNA (*p* = 0.01). Significant correlation between p53 and PCNA in basal and suprabasal layers of dentigerous and Gorlin cysts (*p* < 0.05).	PCNA and p53 expressions in radicular and dentigerous cysts show similar characteristics despite different origins. Different expression patterns in odontogenic keratocysts and Gorlin cysts suggest different growth patterns. Further studies are needed to investigate the role of inflammation in these lesions.
Gaballah et al. (2010) [56]	To investigate the immunohistochemical expression of P53 protein in odontogenic cysts.	Study Type: Immunohistochemical study. Sample Size: 16 radicular cysts (RCs), 11 odontogenic keratocysts (OKCs), 3 dentigerous cysts (DCs). Age Range: Mean age: 35.2 ± 16.5 years. Country/Region: Egypt.	Monoclonal mouse antibody to p53.	Histopathological analysis using hematoxylin–eosin staining.	P53 positive cases: 81.8% OKC, 33.3% DC, 0% RC. P53 expression seen in basal and parabasal cells of epithelial lining.	Data were analyzed using SPSS 10 software. No specific statistical values were provided.	High P53 expression in OKC suggests greater proliferative activity, supporting reclassification as keratocystic odontogenic tumor (KCOT). Low or no P53 expression in RC and DC indicates lower proliferative activity.
Chandrangsu et al. (2016) [57]	To characterize the expression of p53, p63, and p73 in KCOTs and the relationship between their expression and KCOT angiogenesis and recurrence.	Study Type: Immunohistochemical study. Sample Size: 39 KCOTs. Age Range: Mean age: 37.1 ± 21.8 years. Country/Region: Thailand.	Monoclonal antibodies specific to human p53, p63, p73, and CD105.	Histopathological analysis using hematoxylin and eosin staining.	p53 expression: 59% of cases. p63 expression: 82% of cases. p73 expression: 66.7% of cases. Mean MVD: 26.7 ± 15.8 per HPF. Significant positive relationships noted for p53, p63, and p73 expression and MVD (*p* < 0.001). Increased expression of p53, p63, and p73 significantly associated with local recurrence (*p* = 0.001, 0.012, and 0.017, respectively).	Fisher’s exact test, Mann–Whitney U test, Spearman’s correlation coefficients, *p* < 0.05 considered statistically significant.	p53, p63, and p73 expression and increased angiogenesis contribute to the locally aggressive and invasive behaviors of KCOTs, supporting their classification as tumors.
Khan et al. (2019) [58]	To evaluate the management and follow-up of an extensive odontogenic keratocyst (OKC) over a 10-year period.	Study Type: Clinical case report. Sample Size: One case of extensive panmandibular OKC. Age Range: 35-year-old female. Country/Region: Saudi Arabia.	Ki-67, bcl-2.	Radiographic examination and histopathological analysis.	Initial treatment: marsupialization led to reduced Ki-67 and bcl-2 expression. Persistent area required curettage, extraction, and Carnoy’s solution application. 10-year follow-up showed complete resolution with no recurrence.	Not applicable.	Marsupialization is effective as an initial treatment for extensive OKC, but additional aggressive treatment may be necessary for complete resolution. Ki-67 and bcl-2 are site-specific markers related to OKC recurrence.
Kadashetti et al. (2020) [59]	To understand the behavior of epithelial cells in pathogenesis and biological aspects of odontogenic keratocyst (OKC) in diagnosis by analyzing the expression of p53, p63, and proliferating cell nuclear antigen (PCNA).	Study Type: Immunohistochemical study. Sample Size: 21 cases of OKCs. Country/Region: India (Maharashtra).	p53, p63, PCNA.	Histopathological analysis using hematoxylin and eosin staining.	p53-positive cells mainly in suprabasal layers. p63- and PCNA-positive cells found throughout the lining epithelium, including basal and suprabasal layers. p63 and PCNA showed higher staining intensity compared to p53.	Significant difference (*p* < 0.01) between basal and suprabasal cells in OKC.	The biological behavior of OKCs may be related to the suprabasal proliferative compartment. High levels of p53, p63, and PCNA suggest that these proteins contribute to the biological profile and possibly the tumorigenesis of OKCs.
Slusarenko da Silva et al. (2021) [8]	To assess the expression of the p53 protein in odontogenic keratocysts (OKCs) compared to dentigerous cysts (DCs) and ameloblastomas (AMBs) and determine whether OKCs behave more like tumors than cysts.	Study Type: Systematic review and meta-analysis. Sample Size: 13 studies included. Country/Region: Brazil, Netherlands.	p53.	Histopathological criteria defined by WHO in 1992, 2005, and 2017.	126 records identified; 13 studies included. OKCs have a 23% higher probability of expressing p53 compared to DCs (*p* < 0.003). OKCs have a 4% higher probability of expressing p53 compared to AMBs (*p* = 0.28). p53 expressed mainly in the suprabasal layer in OKCs.	Risk Difference (RD) for OKCs vs. DCs: 0.23 [−0.39, −0.08], *p* < 0.003. RD for OKCs vs. AMBs: 0.04 [−0.11, 0.03], *p* = 0.28. Significant heterogeneity among studies (I^2^ = 78% for OKCs vs. DCs, I^2^ = 27% for OKCs vs. AMBs).	OKCs are more likely to express p53, indicating a behavior more like tumors rather than cysts. The classification of OKCs as keratocystic odontogenic tumors (KCOTs) should be reconsidered.
Yanatatsaneejit et al. (2015) [60]	To clarify the association between the p53 polymorphism at codon 72 and susceptibility to the sporadic keratocystic odontogenic tumor (KCOT).	Study Type: Case–control study. Sample Size: 100 KCOT samples and 160 healthy controls. Age Range: Average age of KCOT patients: 32.23 years; Average age of healthy controls: 32.51 years. Country/Region: Thailand.	Polymerase chain reaction–restriction fragment length polymorphism (PCR-RFLP), confirmed by direct sequencing.	Histopathological confirmation of KCOT.	Genotype frequencies in controls: Pro/Pro (23.8%), Arg/Pro (49.4%), Arg/Arg (26.9%). Genotype frequencies in KCOT cohort: Pro/Pro (43.0%), Arg/Pro (39.0%), Arg/Arg (18.0%). p53 Pro allele associated with increased KCOT risk (OR = 1.77, 95% CI = 1.22–2.59, *p* = 0.0024). Sex-adjusted OR = 1.71 (95% CI = 1.17–2.50, *p* = 0.0046). p53 Pro homozygous associated with twofold KCOT risk (adjusted OR = 2.17, 95% CI = 1.23–3.84, *p* = 0.0062).	OR = 1.77 (95% CI = 1.22–2.59, *p* = 0.0024); sex-adjusted OR = 1.71 (95% CI = 1.17–2.50, *p* = 0.0046); adjusted OR = 2.17 (95% CI = 1.23–3.84, *p* = 0.0062).	The C/C genotype of the p53 gene codon 72 increases the risk of developing sporadic KCOT and may be a useful tool for screening and diagnostic purposes.
Varsha et al. (2014) [61]	To investigate the expression of p63 protein in odontogenic keratocyst (OKC), solid ameloblastoma, unicystic ameloblastoma, and follicular tissue, and compare their proliferative activity and biological behavior.	Study Type: Immunohistochemical study. Sample Size: 12 OKCs, 12 solid ameloblastomas, 14 unicystic ameloblastomas, 10 follicular tissues. Country/Region: India (Bangalore, Karnataka).	Anti-p63 polyclonal antibody and super sensitive polymer HRP detection system.	Histopathological analysis using hematoxylin and eosin staining.	p63 expression in suprabasal compartment of OKC equivalent to central neoplastic cells of solid ameloblastoma and unicystic ameloblastoma type 3. Significant difference in p63 expression between OKC and unicystic ameloblastoma type 1, and between solid ameloblastoma and unicystic ameloblastoma type 1. Higher expression of p63 in OKC correlates with aggressive behavior.	Significant difference (*p* < 0.05) in p63 expression between OKC vs unicystic ameloblastoma type 1, and solid ameloblastoma vs unicystic ameloblastoma type 1.	Higher p63 expression in OKC suggests aggressive behavior, supporting the classification as keratocystic odontogenic tumor. Different expression patterns among the lesions can guide treatment modalities and prognosis.
Sajeevan et al. (2014) [62]	To assess and compare the expression of p53 and PCNA in the lining epithelium of odontogenic keratocyst (OKC) and periapical cyst (PA).	Study Type: Retrospective, immunohistochemical study. Sample Size: 10 OKCs and 10 PAs.	p53, PCNA.	Histopathological examination using hematoxylin and eosin staining.	OKC showed 100% PCNA expression; PA showed 60% PCNA expression. OKC showed 60% p53 expression; PA showed 10% p53 expression. PCNA staining intensity was more significant than p53 in both OKC and PA. Suprabasal cells in OKC showed more intense staining than basal cells with both antibodies	Chi-square test showed significant differences in staining intensity (*p* = 0.013 for PCNA vs. p53 in OKC).	OKC shows significant proliferative activity compared to PA using PCNA and p53. PCNA staining is more intense than p53 in both OKC and PA, indicating higher proliferative potential in OKC.
Razavi et al. (2014) [63]	To analyze the clinicopathological and immunohistochemical features of primary and recurrent keratocystic odontogenic tumors (KCOTs), focusing on p53 expression to identify markers predictive of recurrence.	Study Type: Descriptive analytic study. Sample Size: 78 KCOT specimens: 52 primary KCOTs and 26 recurrent KCOTs. Age Range: Mean age: Primary KCOTs: 32.15 ± 16.10 years; Recurrent KCOTs: 27.23 ± 13.04 years. Country/Region: Isfahan, Iran.	Anti-p53 monoclonal antibody.	Histopathological examination using hematoxylin and eosin staining.	No significant differences in age, gender, or anatomical location between primary and recurrent KCOTs. Histopathological features like epithelial budding (*p* = 0.001), daughter cysts (*p* = 0.013), and odontogenic rests (*p* = 0.036) were more common in recurrent KCOTs. p53 expression was significantly higher in recurrent KCOTs (*p* = 0.041).	T-test, Chi-square, and Fisher’s exact tests showed significant differences in histopathological features and p53 expression between primary and recurrent KCOTs.	Predictive factors for KCOT recurrence include epithelial budding, daughter cysts, and odontogenic rests. p53 expression at diagnosis can help predict recurrence.
Chandrashekar et al. (2020) [64]	To investigate the clinical behavior of odontogenic keratocyst (OKC) by evaluating p53, MDM2 expression, and AgNOR staining, and to ascertain if these markers correlate with clinical outcomes and recurrence tendency.	Study Type: Retrospective, immunohistochemical study. Sample Size: 21 histologically confirmed cases of recurrent and non-recurrent OKCs. Country/Region: India (Manipal, Karnataka).	p53 and MDM2, AgNOR staining.	Histopathological examination using hematoxylin and eosin staining.	Recurrent OKCs showed higher expression of MDM2 and AgNOR compared to non-recurrent cases. Recurrent cases displayed histopathological features such as epithelial budding, daughter cysts, and odontogenic rests. Significant difference in MDM2 and AgNOR staining between recurrent and non-recurrent groups (*p* = 0.001). There is no significant difference in p53 scores between recurrent and non-recurrent groups.	Mann–Whitney U-test showed significant differences in MDM2 and AgNOR staining (*p* = 0.001), and significant correlation between p53 and MDM2, and AgNOR and MDM2.	Higher expression of MDM2 and AgNOR in recurrent lesions indicates their potential use in predicting recurrence and guiding additional surgical interventions to improve prognosis.
Deyhimi et al. (2012) [65]	To evaluate the quantity and intensity of the expression of p53 protein and transforming growth factor alpha (TGF-alpha) in odontogenic keratocyst (OKC) and orthokeratinized odontogenic cyst (OOC) to compare their biological behavior.	Study Type: Cross-sectional, descriptive analytic study. Sample Size: 15 OKC and 15 OOC. Country/Region: Iran.	Monoclonal anti-p53 and anti-TGF-alpha antibodies.	Histopathological examination using hematoxylin and eosin staining.	p53: No significant difference in the basal layer (*p* = 0.076), significant in the parabasal layer (*p* = 0.003). TGF-alpha: No significant difference in the basal layer (*p* = 0.284), significant in the parabasal layer (*p* = 0.015). Higher expression of p53 and TGF-alpha in OKC compared to OOC, suggesting higher carcinomatous potential in OKC.	Mann–Whitney and Wilcoxon tests; significant differences noted with *p* < 0.05.	OKC shows higher expression of p53 and TGF-alpha than OOC, indicating a higher theoretical potential for carcinomatous changes in OKC.
Ogden et al. (1992) [66]	To assess p53 protein expression in a range of odontogenic cysts, including developmental and inflammatory origins.	Study Type: Immunohistochemical study. Sample Size: 12 radicular cysts, 12 dentigerous cysts, 12 odontogenic keratocysts (OKC). Country/Region: Scotland.	Polyclonal antibody CM-1 and standard immunoperoxidase technique.	Histopathological examination using hematoxylin and eosin staining.	p53 expression detected in 5 of 12 OKCs, but not in radicular or dentigerous cysts. p53-positive cells actively dividing, similar regions positive for PCNA. No significant difference in clinical characteristics or recurrence between p53-positive and p53-negative OKCs. p53-positive OKCs lacked features like cholesterol clefts, hyaline bodies, or satellite cysts.	Not provided.	Increased p53 expression in some OKCs suggests higher epithelial activity and potential association with Gorlin Goltz syndrome. p53 expression may be indicative of malignant potential within OKC linings.
Aldahash (2023) [67]	To systematically review and perform a meta-analysis on the expression of p53 in odontogenic lesions, including odontogenic keratocyst (OKC), dentigerous cyst (DC), and ameloblastoma (AMB).	Study Type: Systematic review and meta-analysis. Sample Size: 18 studies included in the meta-analysis. Country/Region: Saudi Arabia.	p53.	Histopathological criteria defined by WHO in 1992, 2005, and 2017.	OKCs have a 23% higher probability of expressing p53 compared to DCs (*p* = 0.003). OKCs have a 4% lower probability of expressing p53 compared to AMBs (*p* = 0.028). p53 expression suggests that OKCs act more like tumors than cysts.	Risk difference (RD) for p53 expression between OKCs and DCs: 0.23 [0.39, 0.08], *p* = 0.003. RD between OKCs and AMBs: −0.04 [−0.11, 0.03], *p* = 0.028.	OKCs exhibit higher p53 expression compared to DCs, indicating a more tumor-like behavior. This supports reclassifying OKCs as keratocystic odontogenic tumors (KCOTs).
Gupta et al. (2019) [68]	To compare the expression pattern of p63 in the epithelium of tooth germ, dentigerous cyst (DC), and ameloblastoma (AB).	Study Type: Descriptive observational study. Sample Size: 30 tooth germs, 30 DCs, 30 ABs. Country/Region: India (Chhattisgarh, Maharashtra).	p63 antibody and Streptavidin–Biotin Detection System HRP-DAB.	Histopathological examination using hematoxylin and eosin staining.	p63 expression in 100% of tooth germs, 100% of DCs, 100% of ABs. Highest p63 labeling index (LI) in ABs, followed by tooth germs, and then DCs. Dense p63 immunolabeling in the basal and parabasal layers of DCs; peripheral cells of ameloblastic follicle in ABs; almost complete epithelium in tooth germs.	Non-significant difference in p63 LI among ABs, DCs, and tooth germs (*p* > 0.05).	p63 plays a role in the differentiation and proliferation of odontogenic epithelial cells. p63 can be used as a prognostic marker for aggressive and invasive odontogenic lesions. Different p63 isoforms may have distinct functions in developing and lesional odontogenic tissues.
Akshatha et al. (2017) [69]	To qualitatively and quantitatively analyze the expression of inducible nitric oxide synthase (iNOS) in the epithelial lining of radicular cysts (RCs), dentigerous cysts (DCs), and odontogenic keratocysts (OKCs) to determine the role of iNOS in their pathogenesis.	Study Type: Qualitative and quantitative immunohistochemical study. Sample Size: 20 RCs, 20 DCs, and 20 OKCs. Country/Region: India (Bengaluru, Karnataka).	Immunohistochemistry using anti-iNOS antibody.	Histopathological examination using hematoxylin and eosin staining.	iNOS-positive cells: OKCs (57.1%), RCs (28.6%), DCs (14.3%). Significant iNOS expression in OKCs (*p* > 0.000) and RCs (*p* > 0.001), but not in DCs. iNOS staining in OKCs: 47.4% showed severe intensity, mainly in basal and parabasal layers; 31.6% of RCs and 21.1% of DCs showed severe intensity. There was no significant difference in staining intensity among the three cyst types.	Mann–Whitney test and contingency coefficient showed statistically significant iNOS expression in OKCs (*p* > 0.000), RCs (*p* > 0.001), no significant values for DCs.	Increased iNOS expression in OKCs may contribute to their aggressive behavior and malignant potential, suggesting a role in bone resorption and accumulation of wild-type p53 protein.
Fatemeh et al. (2017) [70]	To assess and compare the expression of the tumor suppressor gene p53 in inflamed and non-inflamed types of odontogenic keratocyst (OKC) and dentigerous cyst (DC).	Study Type: Immunohistochemical study. Sample Size: 34 OKCs (18 inflamed, 16 non-inflamed), 31 DCs (16 inflamed, 15 non-inflamed), 14 dental follicles. Country/Region: Iran.	Monoclonal mouse antihuman p53 antibody.	Histopathological examination using hematoxylin and eosin staining.	Mean percentage of p53-positive cells: dental follicles (0.7%), non-inflamed OKCs (5.4%), inflamed OKCs (17.3%), non-inflamed DCs (1.2%), and inflamed DCs (2.2%). Significant differences between all groups (*p* < 0.05) except between inflamed and non-inflamed DCs, and between dental follicles and non-inflamed DCs.	Kruskal–Wallis H and Mann–Whitney U tests showed significant differences (*p* < 0.05) between the groups for p53 expression.	p53 expression is higher in OKCs than in DCs, suggesting different growth mechanisms. Inflammation increases p53 expression in OKCs, indicating a potential role in their aggressive behavior.
Seyedmajidi et al. (2013) [12]	To evaluate the expression of p53 and PCNA in keratocystic odontogenic tumors (KCOTs) compared with radicular cysts (RCs), dentigerous cysts (DCs), and calcifying cystic odontogenic tumors (CCOTs).	Study Type: Immunohistochemical study. Sample Size: 25 RCs, 23 DCs, 23 KCOTs, and 23 CCOTs. Country/Region: Iran.	p53, PCNA.	Histopathological examination using hematoxylin and eosin staining.	Highest p53 expression in the basal layer of RC and suprabasal layer of KCOT. Highest PCNA expression in the suprabasal layer of KCOT. Significant differences in p53 expression in basal and suprabasal layers, and PCNA expression in the suprabasal layer between cysts. No significant difference in PCNA expression in the basal layer among the cysts.	Kruskal–Wallis test (*p* = 0.008 for p53 in basal; *p* = 0.031 for p53 in suprabasal; *p* = 0.009 for PCNA in suprabasal). Wilcoxon signed rank test (*p* = 0.007 for p53 in basal layer RC; *p* = 0.024 for p53 in basal layer DC; *p* = 0.025 for p53 in basal layer KCOT).	The high expression of PCNA in the suprabasal layer of KCOT supports its neoplastic nature and tendency for recurrence. p53 expression in the basal layer of RC indicates an inflammatory response.

### 4.4. Matrix Metalloproteinases (MMPs) and Their Role

Matrix metalloproteinases (MMPs), particularly MMP-2 and MMP-9, play crucial roles in the development and progression of odontogenic lesions such as dentigerous cysts (DCs) and odontogenic keratocysts (OKCs) [71,72,73]. These enzymes are instrumental in the breakdown of extracellular matrix components, contributing to the rapid growth and potential recurrence of these cysts and tumors by enhancing their invasiveness. Genetic studies have linked specific gene variations of MMPs to the aggressive nature of lesions such as ameloblastomas and keratocystic odontogenic tumors (KCOTs), suggesting the potential for therapies targeting these genetic traits [72]. Additionally, the presence of MMP-7 and MMP-9 has been associated with more aggressive behavior in keratocysts related to nevoid basal cell carcinoma syndrome (NBCCS), indicating these enzymes as potential markers for distinguishing between syndromic and non-syndromic lesions [73].

The examination of MMPs in lesions like DCs and OKCs not only deepens our understanding of these conditions but also reveals how these enzymes contribute to their pathology, leading to the development of targeted treatments based on the lesions’ molecular and genetic characteristics. For instance, studies have specifically linked MMP-9 to the aggressive behavior of odontogenic keratocysts, suggesting that MMP inhibitors could serve as effective therapeutic agents. Clinical case reports have demonstrated that the local application of MMP inhibitors can significantly reduce the invasiveness of these lesions, supporting their use as adjunct therapies alongside conventional surgical methods [74,75]. This practical application highlights how understanding MMP activity can lead to more targeted and effective treatment approaches, demonstrating the direct impact of molecular insights on improving clinical outcomes.

Recent studies have provided significant insights into the role of MMPs in odontogenic lesions. Ortiz-García et al. analyzed the expression levels and proteolytic activities of MMP-2 and MMP-9 in various odontogenic lesions, finding that both enzymes showed higher proteolytic activity in cystic and tumor lesions compared to dental follicles, highlighting their role in the growth and development of these lesions [71]. Aloka et al. conducted a pilot study on the gene polymorphisms of MMP-2 and MMP-9 in aggressive and nonaggressive odontogenic lesions. They found significant associations between specific polymorphisms and the aggressiveness of lesions such as ameloblastomas and KCOTs, indicating that these genetic traits could guide the development of targeted therapies [72]. Furthermore, Loreto et al. examined the expression of MMP-7 and MMP-9 in NBCCS-related, recurrent, and sporadic keratocysts. Their findings suggested that higher expressions of these MMPs in NBCCS-OKCs correlate with the more aggressive and recurrent nature of these lesions, emphasizing the potential of MMPs as therapeutic targets [73].

The practical applications of these findings are significant. The use of MMP inhibitors as adjunct therapies has been shown to reduce the invasiveness of odontogenic lesions, offering a less invasive alternative to conventional surgical methods. This integration of molecular insights into clinical practice can enhance the efficacy of treatments tailored to specific genetic profiles, ultimately improving patient outcomes [74,75].

In summary, MMPs offer key insights into the biological processes underlying odontogenic lesions. This knowledge not only aids in diagnosis but also informs the development of targeted therapeutic interventions, promising new, more effective ways to manage these conditions and improve patient outcomes [71,72,73,74,75] (Table 4).

**Table 4:** Matrix metalloproteinases (MMP-2, MMP-7, and MMP-9).

Authors	Objective	Study Details	Marker Identification Method	Cyst/Tumor Diagnosis Method	Results	Statistical Estimates	Conclusion
Ortiz-García et al. (2022) [71]	To compare the expression level and proteolytic activities of MMP-2 and MMP-9 in dental follicles (DFs), dentigerous cysts (DCs), odontogenic keratocysts (OKCs), and unicystic ameloblastomas (UAs).	Study Type: Immunohistochemical and proteolytic activity study. Sample Size: 7 DFs, 8 DCs, 8 OKCs, and 8 UAs. Country/Region: Mexico.	Antibodies for MMP-2 and MMP-9, Western blot analysis.	Histopathological examination using hematoxylin and eosin staining.	MMP-2: Similar expression in all tissues, but higher activity in cystic and tumor lesions compared to DF. MMP-9: Higher expression and activity in DC, OKC, and UA compared to DF. No significant differences in MMP-2 or MMP-9 expression and activity between cystic and tumor lesions.	not specified.	MMP-2 and MMP-9 play a role in the development of DC, OKC, and UA. Their increased activity suggests involvement in lesion growth, but they do not differentiate between cystic and tumor lesions.
Aloka et al. (2019) [72]	To investigate the association of matrix metalloproteinase 2 (MMP2) and matrix metalloproteinase 9 (MMP9) gene polymorphisms with the aggressiveness of ameloblastomas, keratocystic odontogenic tumors (KCOTs), and dentigerous cysts (DCs).	Study Type: Pilot case–control study. Sample Size: 15 ameloblastomas, 11 KCOTs, 13 DCs, and 106 controls. Country/Region: India (Kerala).	PCR-restriction fragment length polymorphism (PCR-RFLP) and sequencing for MMP2 and MMP9 gene polymorphisms.	Histopathological confirmation using WHO (2005) criteria.	Ameloblastomas showed a higher frequency of the MMP9 rs3918242 T allele (*p* = 0.05). Significant differences in MMP2 rs243865 genotype (*p* = 0.046) and allele frequency (*p* = 0.03; OR = 2.06) between cases and controls. KCOT samples showed significant differences in genotype (*p* = 0.01) and allele (*p* = 0.01; OR = 3.42) frequencies compared to controls.	Chi-square analysis revealed significant associations between MMP2 rs243865 and MMP9 rs3918242 polymorphisms and odontogenic lesions (*p* < 0.05).	MMP2 rs243865 polymorphism is associated with increased aggressiveness in ameloblastomas and KCOTs. MMP9 rs3918242 polymorphism may contribute to the aggressive behavior of ameloblastomas.
Loreto et al. (2022) [73]	To analyze the immunohistochemical expression of MMP-7, MMP-9, α-SMA, desmin, and caldesmon in odontogenic keratocysts (OKCs) associated with NBCCS compared to recurrent and sporadic keratocysts.	Study Type: Immunohistochemical study. Sample Size: 40 patients (23 males, 17 females) with OKCs: 19 sporadic OKCs, 9 recurrent OKCs, and 12 NBCCS-associated OKCs. Age Range: Mean age: 32 ± 8.7 years. Country/Region: Italy.	antibodies for MMP-7, MMP-9, α-SMA, desmin, and caldesmon.	Histopathological examination using hematoxylin and eosin staining.	Significantly increased expression of α-SMA, caldesmon, MMP-7, and MMP-9 in NBCCS-OKCs compared to non-syndromic OKCs (*p* < 0.001). Desmin showed a non-significant increase in expression in non-syndromic OKCs compared to NBCCS-OKCs. NBCCS-OKCs showed greater distribution of myofibroblasts (MFs) compared to other OKC subtypes.	Statistical significance with *p*-values < 0.05 for α-SMA, caldesmon, MMP-7, and MMP-9 expressions in NBCCS-OKCs.	Different expression patterns of MMP-7, MMP-9, α-SMA, desmin, and caldesmon suggest distinct biological behaviors of OKC subtypes. NBCCS-OKCs showed higher levels of these markers, indicating greater aggressiveness and recurrence potential. Further studies are needed to correlate these findings with clinical behavior.
Suojanen et al. (2014) [74]	To evaluate the expression of matrix metalloproteinases (MMPs) 8, 9, 25, and 26, and tissue inhibitor of metalloproteinases-1 (TIMP-1) in dentigerous cysts (DCs) and healthy dental follicles (HDFs).	Study Type: Immunohistochemical study. Sample Size: 10 DCs and 10 HDFs. Age Range: Mean age: DC group: 39 years; HDF group: 22 years. Country/Region: Finland.	Immunohistochemistry using polyclonal antibodies for MMPs and TIMP-1.	Histopathological examination using hematoxylin and eosin staining.	MMP-8: Slightly more expressed in DC epithelium than in HDF epithelium, but not statistically significant (*p* = 0.255). MMP-9, MMP-25, MMP-26, and TIMP-1: No significant differences in expression between DCs and HDFs. TIMP-1: Strong positivity in both DCs and HDFs, no difference between groups (*p* = 1.000).	Fisher’s non-parametric exact test: No significant differences found in MMP or TIMP-1 expressions between groups.	Differences in MMP expression cannot solely explain dentigerous cyst expansion, suggesting involvement of other osteolytic mechanisms.
Kuźniarz et al. (2021) [75]	To elucidate the role of MMP-2, MMP-9, and their endogenous inhibitors TIMP-1 and TIMP-2 in the pathogenesis of maxillofacial cystic lesions.	Study Type: Immunohistochemical and gelatin zymography study. Sample Size: 20 with radicular cysts (RC), 7 with retention cysts (RtC), and 3 with dentigerous cysts (DC). Age Range: 18–66 years (median age: 43.2). Country/Region: Poland.	Gelatin zymography for MMP-2 and MMP-9, ELISA for TIMP-1 and TIMP-2.	Clinical examination, CT, panoramic X-ray, histopathological examination.	MMP-9 activity and MMP-9/TIMP-1 ratio were higher in RC fluid compared to RtC fluid. No significant differences in MMP-2 activity in the wall of RtCs compared to DCs. TIMP-1 serum levels were lower in RC patients compared to DC and RtC patients, but not statistically significant.	Kruskal–Wallis test followed by Dunn’s post-hoc test; significance considered at *p* < 0.05.	MMP-9 plays a significant role in the pathogenesis of RCs, while MMP-2 activity is less significant in RtCs. MMP-9 could serve as a biomarker for RC etiology. Further studies are needed to confirm these findings and their clinical implications.
de Andrade Santos et al. (2011) [76]	To compare the immunohistochemical expression of NF-κB, MMP-9, and CD105 in odontogenic keratocysts (OKCs), dentigerous cysts (DCs), and radicular cysts (RCs).	Study Type: Immunohistochemical study. Sample Size: 20 OKCs, 20 DCs, and 20 RCs.	Immunohistochemistry for NF-κB, MMP-9, and CD105.	Histopathological examination using hematoxylin and eosin staining.	NF-κB LI higher in OKCs than in DCs and RCs (*p* < 0.001). MMP-9 expression score 2 in epithelial component: OKCs (90%), DCs (70%), and RCs (65%; *p* = 0.159). No significant difference in NF-κB LI according to MMP-9 expression in epithelial lining (*p* = 0.282). Highest MMP-9 expression in fibrous capsule: OKCs (*p* = 0.100). MMP-9 expression in vessels: score 2 in OKCs (80%) and RCs (50%), score 1 in DCs (75%; *p* = 0.002). Mean microvessel count: RCs (16.9), DCs (12.1), OKCs (10.0; *p* = 0.163). No significant difference in microvessel count according to MMP-9 expression between groups (*p* = 0.689).	*p*-values: NF-κB LI (*p* < 0.001), MMP-9 expression in epithelial component (*p* = 0.159), NF-κB LI according to MMP-9 expression (*p* = 0.282), MMP-9 expression in fibrous capsule (*p* = 0.100), MMP-9 expression in vessels (*p* = 0.002), mean microvessel count (*p* = 0.163), microvessel count according to MMP-9 expression (*p* = 0.689).	The more aggressive biological behavior of OKCs is related to higher expression of MMP-9 and NF-κB. Differences in the biological behavior of the lesions studied do not seem to be associated with the angiogenic index.
Ribeiro et al. (2011) [77]	To evaluate the immunohistochemical expression of matrix metalloproteinases (MMPs) 1, 2, 7, 9, and 26 in calcifying cystic odontogenic tumor (CCOT).	Study Type: Immunohistochemical study. Sample Size: 10 cases of CCOT. Country/Region: Brazil.	Immunohistochemistry using monoclonal antibodies for MMP-1, MMP-2, MMP-7, MMP-9, and MMP-26.	Histopathological examination using hematoxylin and eosin staining.	MMP-1, MMP-7, and MMP-9: score 2 in 100% of cases. MMP-2: score 0 in 90% of cases. MMP-26: varied immunostaining. All MMPs except MMP-2 were expressed in the stroma, indicating their role in ECM degradation and tumor growth. Diffuse immunoreactivity observed for MMPs 1, 7, 9, and 26 in parenchymal cells, while MMP-2 showed a focal pattern.	Descriptive analysis of immunohistochemical expression, no specific statistical tests mentioned.	MMPs 1, 7, 9, and 26 contribute to tumor growth and expansion in CCOT. The presence of these MMPs in stromal cells highlights their involvement in ECM degradation and active tumor growth. Further studies using additional techniques are recommended to better understand the role of these MMPs in CCOT.

### 4.5. Cytokeratins and Other Markers

Cytokeratins (CKs) and markers such as survivin, E-cadherin, CD138, and CD38 are critical for understanding the development, behavior, and diagnosis of odontogenic lesions, including cysts and tumors. The expression patterns of these markers provide valuable information about the biological behavior of these lesions, influencing their management and prognosis [6,78,79,80].

The presence of these markers in specific lesions such as central adenoid basal (CAB), keratocystic odontogenic tumor (KCOT), dentigerous cyst (DC), and radicular cyst (RC) is crucial for accurate diagnosis and assessment of aggressiveness. For instance, increased survivin expression, typically associated with tumor survival and resistance to apoptosis, has been targeted in recent therapeutic trials with survivin inhibitors, showcasing a direct clinical application of these biomarkers in enhancing treatment efficacy. This emphasizes how differential expression of markers like survivin can inform treatment choices and potentially improve clinical outcomes by targeting specific molecular pathways involved in lesion survival and growth [6,78].

Research into markers like syndecan-1 (CD138) and CD56 (NCAM) has also revealed their roles in tumor development and their potential to help distinguish between cystic and tumorous odontogenic lesions. Studies have shown strong CD138 expression in KCOTs and dentigerous cysts, aiding in differentiating these from other lesions. Additionally, CD56 has been noted for its aberrant expression in KCOTs, particularly in syndromic cases, helping to differentiate these from orthokeratinized odontogenic cysts (OOCs) and other similar lesions [79,80].

Investigations into CK expression have emphasized its significance in differentiating between various odontogenic lesions, aiding in the identification of their histopathological features and suggesting different underlying causes for conditions such as OOCs and epithelial dysplasia cysts (EDCs). For example, CK10 and CK19 expression patterns have been useful in distinguishing OOCs from epidermoid cysts (EDCs) and odontogenic keratocysts (OKCs), which is crucial for accurate diagnosis and management. Furthermore, the expression of CK14 and CK18 has been explored in various lesions, revealing differences that help understand their pathogenesis and behavior [81,82,83].

The analysis of these markers not only enriches diagnostic capabilities but also points toward potential new treatments, enhancing the ability to predict and manage the outcomes of odontogenic cysts and tumors more effectively. Understanding these markers’ roles in lesion pathophysiology aids clinicians in tailoring therapeutic approaches based on specific diagnostic and prognostic data, ultimately leading to more effective and personalized patient care [80,83].

In conclusion, the study of CKs, survivin, E-cadherin, CD138, and CD38 provides a deeper understanding of the molecular mechanisms underlying odontogenic lesions. This knowledge is instrumental in developing more accurate diagnostic tools and effective therapeutic strategies, leading to improved patient outcomes in the management of odontogenic cysts and tumors [6,78,79,80,81,82,83] (Table 5).

**Table 5:** Cytokeratins (CK7, CK10, CK13, CK14, CK17, CK18, CK19, survivin, E-cadherin, CD138, CD56, and CD38).

Authors	Objective	Study Details	Marker Identification Method	Cyst/Tumor Diagnosis Method	Results	Statistical Estimates	Conclusion
Özcan et al. (2015) [6]	To investigate the expressions of survivin, E-cadherin, CD138, and CD38 in cystic ameloblastoma, keratocystic odontogenic tumor (KCOT), dentigerous cyst (DC), and radicular cyst (RC), and their potential diagnostic usage.	Study Type: Immunohistochemical study. Sample Size: 5 RCs, 5 DCs, 5 KCOTs, 5 cystic ameloblastomas. Country/Region: Turkey.	Immunohistochemistry using antibodies for survivin, E-cadherin, CD138, and CD38.	Histopathological examination using hematoxylin and eosin staining.	Cystic ameloblastomas and KCOTs showed diffuse and strong nuclear survivin expression. No specific survivin immunoreactivity in DCs and RCs. E-cadherin expression stronger in DCs and RCs compared to other lesions. CD138 expression prominent in stromal cells of cystic ameloblastomas, gradually decreased in other lesions. All cases were negative for CD38.	Not provided.	Loss of E-cadherin expression in epithelial cells, strong CD138 expression in stromal cells, and strong nuclear survivin expression in cystic ameloblastomas and KCOTs are characteristic findings, suggesting a role in their aggressiveness and pathogenesis.
Etemad-Moghadam et al. (2017) [78]	To assess the immunohistochemical expression of CD138 (syndecan-1) in adenomatoid odontogenic tumor (AOT), ameloblastic fibroma (AF), and odontogenic myxoma (OM), and to compare it with ameloblastoma and keratocystic odontogenic tumor (KCOT).	Study Type: Immunohistochemical study. Sample Size: 7 AOTs, 5 OMs, 7 AFs, 29 KCOTs, and 10 ameloblastomas. Country/Region: Iran.	Immunohistochemistry using monoclonal antibody against syndecan-1 (CD138).	Histopathological examination using hematoxylin and eosin staining.	Syndecan-1 expressed in all samples except OMs. Significant differences in percentage and intensity of syndecan-1 expression among the studied OTs (*p* < 0.001). Pairwise comparisons showed significant difference only between OMs and each of the other tumors.	Kruskal–Wallis test followed by Bonferroni analysis for comparisons (*p* < 0.05).	Syndecan-1 may be involved in the pathogenesis of AOT, AF, KCOT, and ameloblastoma. However, its effect on clinical aggressiveness appears limited. Negative immunoexpression in OM requires further investigation.
Vera-Sirera et al. (2015) [79]	To investigate the immunohistochemical expression of neural cell adhesion molecule (NCAM, CD56) in keratin-producing odontogenic cysts (KPOCs) including keratocystic odontogenic tumors (KCOTs) and orthokeratinized odontogenic cysts (OOCs).	Study Type: Immunohistochemical study. Sample Size: 12 OOCs and 46 KCOTs (40 non-syndromic KCOTs, 6 syndromic KCOTs associated with nevoid basal cell carcinoma syndrome (NBCS)). Country/Region: Spain.	Immunohistochemistry using monoclonal antibody NCL-CD56-504 (clone CD564).	Histopathological examination using hematoxylin and eosin staining.	NCAM expression: 0% in OOCs, 36.95% in KCOTs. Focal and heterogeneous NCAM expression in 36.95% of KCOTs, especially at the basal cell level, basal budding areas, and basal cells of daughter cysts. Higher NCAM expression in syndromic KCOTs (66.66%) compared to non-syndromic KCOTs (30%). Significant difference in NCAM expression between OOCs and KCOTs (*p* = 0.012). No association between NCAM expression and lesion recurrence.	Firth’s logistic regression test; significant difference with *p* < 0.05 for NCAM expression between OOCs and KCOTs.	Aberrant NCAM expression in KCOTs, especially in syndromic KCOTs, suggests a role in pathogenesis and potential impact on lesional recurrence. Further studies with homogeneously treated series are needed to confirm these findings.
Jaafari-Ashkavandi et al. (2014) [80]	To examine the expression of CD56 in ameloblastomas, dentigerous cysts, keratocystic odontogenic tumors (KCOTs), adenomatoid odontogenic tumors (AOTs), orthokeratinized odontogenic cysts, calcifying odontogenic cysts (COCs), and glandular odontogenic cysts (GOCs).	Study Type: Cross-sectional, analytical immunohistochemical study. Sample Size: 22 ameloblastomas (14 solid, 8 unicystic), 13 dentigerous cysts, 10 KCOTs, 4 AOTs, 3 orthokeratinized odontogenic cysts, 3 COCs, 1 GOC. Country/Region: Iran.	Immunohistochemistry using anti-CD56 antibody.	Histopathological examination using hematoxylin and eosin staining.	CD56 expression: 91% in ameloblastomas, 75% in AOTs, 40% in KCOTs, 100% in GOCs. No CD56 expression in dentigerous cysts, COCs, orthokeratinized odontogenic cysts. Significant difference in CD56 expression between ameloblastoma and dentigerous cysts, and between KCOT and orthokeratinized odontogenic cysts.	Chi-square test; significant differences with *p* < 0.05 in CD56 expression between specific groups.	CD56 expression is limited to more aggressive cysts and tumoral lesions, suggesting it can be a useful marker for distinguishing between cystic and tumoral odontogenic lesions.
Al-Otaibi et al. (2013) [84]	To analyze the expression profile of syndecan-1 (SD-1) immunohistochemically in ameloblastomas and common odontogenic cysts.	Study Type: Immunohistochemical study. Sample Size: 32 ameloblastomas, 26 keratocystic odontogenic tumors (KCOTs), 21 dentigerous cysts.	Immunohistochemistry using monoclonal antibody against SD-1 (CD138).	Histopathological examination using hematoxylin and eosin staining.	SD-1 expression in epithelial and stromal elements significantly associated with lesion extension and involvement of adjacent structures (*p* = 0.025). Higher SD-1 expression in stellate reticulum cells than ameloblasts (*p* < 0.0001). Significant difference in epithelial staining among ameloblastomas, KCOTs, and dentigerous cysts (*p* < 0.0001). Mean rank scores (Kruskal–Wallis test): ameloblastomas significantly lower than KCOTs and dentigerous cysts; non-significant comparison between KCOT and dentigerous cyst groups.	*p*-values: lesion extension and involvement (*p* = 0.025), stellate reticulum cells vs. ameloblasts (*p* < 0.0001), epithelial staining among groups (*p* < 0.0001).	SD-1 expression in stromal cells of ameloblastoma, KCOT, and dentigerous cyst is associated with poor prognosis.
Krishnan et al. (2023) [85]	To determine if specific patterns of CK14 and Bcl-2 staining can assist in diagnosing OKCs with altered epithelial features and provide insights into their aggressive nature.	Study Type: Immunohistochemical study.	Immunohistochemistry for CK14 and Bcl-2.	Histopathological examination.	CK14 expression: Restricted to basal and suprabasal layers near satellite cysts and areas with subepithelial split. Entire epithelial lining showed CK14 expression in areas of inflammation and after marsupialization. Bcl-2: Typical basal/suprabasal staining lost in areas of inflammation, and intensity decreased in OKCs after marsupialization.	Not specified.	Specific CK14 and Bcl-2 staining patterns could aid in diagnosing OKCs with altered epithelial features and provide insights into their aggressive behavior. Proper recognition and diagnosis are essential for treatment planning due to therapeutic consequences and high recurrence rates.
Padmapriya et al. (2020) [81]	To compare the cytokeratin expressions of CK10 and CK19 among orthokeratinized odontogenic cysts (OOCs), epidermoid cysts (EDCs), and odontogenic keratocysts (OKCs) by immunohistochemical study.	Study Type: Immunohistochemical study. Sample Size: 10 OOCs, 10 EDCs, 10 OKCs. Country/Region: India.	Immunohistochemistry using CK10 and CK19 markers.	Histopathological examination using hematoxylin and eosin staining.	CK10 expression: 100% in OOCs and EDCs, 50% in OKCs. CK19 expression: 40% in OOCs, 30% in EDCs, 80% in OKCs. Significant difference in CK10 expression between OKCs and OOCs/EDCs (*p* = 0.009). CK19 expression significant between EDCs and OKCs (*p* = 0.028), but not between OOCs and EDCs or OOCs and OKCs.	*p*-values: CK10 expression (*p* = 0.002), CK19 expression (*p* = 0.061).	CK10 expressions in OOCs and EDCs were nearly identical, indicating OOCs might not be distinguished from EDCs histologically. CK19 expression showed no significant differences between OOCs and EDCs or OOCs and OKCs, implying OOCs resemble both EDCs and OKCs.
Sheethal et al. (2019) [82]	To present a rare case of an odontogenic keratocyst (OKC) arising in the maxillary sinus and discuss its clinical, radiographic, and histopathological features.	Study Type: Case report. Sample Size: Single case. Age Range: 15-year-old female. Country/Region: India (Bengaluru, Karnataka).	Not applicable (case report).	Histopathological examination.	Patient presented with pain and pus discharge in the upper left back teeth region. Radiographs revealed an ill-defined radiolucent lesion associated with an impacted third molar in the maxillary sinus. Histopathological examination showed parakeratinized stratified squamous epithelium, nuclear hyperchromatism, and palisading of basal cells. The lesion was diagnosed as OKC in the maxillary sinus.	Not applicable.	OKC in the maxillary sinus is rare and can be mistaken for other lesions like sinusitis or antral polyps. Proper recognition and diagnosis are crucial due to its aggressive behavior and high recurrence rate. Long-term follow-up is necessary to monitor for recurrence.
Hoshino et al. (2015) [83]	To perform an immunohistochemical analysis of cytokeratins (CKs) and langerin to examine differences in the lining epithelium of dermoid cysts (DMCs), epidermoid cysts (EDMCs), orthokeratinized odontogenic cysts (OOCs), and keratocystic odontogenic tumors (KCOTs).	Study Type: Immunohistochemical study. Sample Size: 7 DMCs, 30 EDMCs, 11 OOCs, 28 KCOTs. Age Range: Mean ages: DMCs (42.9 years), EDMCs (40.8 years), OOCs (36.3 years), KCOTs (33.5 years). Country/Region: Japan.	Immunohistochemistry using antibodies against CK10, CK13, CK14, CK16, CK17, CK19, and langerin.	Histopathological examination using hematoxylin and eosin staining.	CK10: Positive in all layers except basal layer in DMCs, EDMCs, and OOCs; negative in KCOTs. CK13 and CK17: Positive in all layers except basal layer in OOCs; negative in DMCs/EDMCs. CK14 and CK16: Positive in all layers in DCs; CK14 positive in all except surface layer in DMCs, EDMCs, OOCs, and KCOTs. CK19: Negative in OOCs; positive in all layers except basal layer in KCOTs. Langerin: Positive in Langerhans cells in the spinous layer of DMCs, EDMCs, and OOCs; rarely positive in KCOTs; negative in DCs.	Not specified.	The immunohistochemical profiles of CKs and langerin in DMCs/EDMCs, OOCs, and KCOTs suggest that these are independent diseases with distinct biological characteristics.
Yamamoto et al. (2013) [86]	To report a case of keratocyst in the buccal mucosa with features of a keratocystic odontogenic tumor (KCOT).	Study Type: Case report. Sample Size: Single case. Age Range: 74-year-old male. Country/Region: Japan.	Immunohistochemistry using antibodies for CK17, CK10, and Ki-67.	Histopathological examination using hematoxylin and eosin staining, clinical and imaging examination.	The patient had a cystic lesion in the right buccal mucosa, initially diagnosed as an epidermoid cyst. Histopathological examination revealed parakeratinized squamous epithelium with palisading basal cells. Immunohistochemistry showed positive CK17 and high Ki-67 labeling, indicating high proliferation potential, and negative CK10. Features were consistent with KCOT.	Not applicable.	The keratocyst in the buccal mucosa showed characteristics of KCOT, suggesting an odontogenic origin. Accurate diagnosis and differentiation from other cystic lesions are crucial for appropriate treatment and management.
Kureel et al. (2019) [87]	To analyze the immunohistochemical (IHC) expression of cytokeratins (CK10, CK13, and CK19) and fibronectin in orthokeratinized odontogenic cyst (OOC), epidermoid cyst (EDC), dermoid cyst (DMC), dentigerous cyst (DC), and keratocystic odontogenic tumor (KCOT) to elucidate the pathogenesis of OOC.	Study Type: Immunohistochemical study. Sample Size: 25 cases for each study group: OOC, EDC, DMC, DC, KCOT. Country/Region: India.	Immunohistochemistry using CK10, CK13, CK19, and fibronectin antibodies.	Histopathological examination using hematoxylin and eosin staining.	CK10: Similar expression in OOC and EDC; negative in DC; positive only in the surface layer of KCOT. CK13: Mild expression in OOC and EDC; intense in KCOT and DC. CK19: Negative to mild in OOC; negative in EDC; positive in DC and KCOT. Fibronectin: Predominantly diffuse non-fibrillar (DN) pattern in EDC, followed by DC, KCOT, and OOC; focal non-fibrillar (FN) not detected in OOC.	Chi-square test and Spearman’s correlation; significant differences in CK10, CK13, CK19, and fibronectin expressions among the study groups (*p* ≤ 0.05).	The IHC profile of OOC is different from DC and KCOT but closer to EDC. CK10 expression in OOC suggests normal orthokeratinization. Strong CK19 expression in DC and KCOT confirms odontogenic origin. OOC likely represents the intraosseous counterpart of EDC.
Bhakhar et al. (2016) [88]	To evaluate the intensity and expression patterns of cytokeratins (CKs) 18 and 19 in odontogenic keratocysts (OKCs), dentigerous cysts (DCs), and radicular cysts (RCs).	Study Type: Immunohistochemical study. Sample Size: 20 OKCs, 20 DCs, 20 RCs. Country/Region: India.	Immunohistochemistry using monoclonal antibodies for CK18 and CK19.	Histopathological examination using hematoxylin and eosin staining.	CK18 expression: 25% in OKCs, 15% in DCs, 10% in RCs, mostly focal with mild intensity. CK19 expression: 75% in OKCs, 100% in DCs and RCs, with intense and “ALL” expression in DCs and RCs, and moderate in OKCs.	Z-test and Pearson’s chi-square test showed significant differences in CK18 and CK19 expressions among the cysts (*p* ≤ 0.05). CK18 expression in OKCs (*p* = 0.001), DCs (*p* = 0.000), and RCs (*p* = 0.000).	CK19 had higher intensity and expression in all cyst types compared to CK18, suggesting CK19 as a valuable diagnostic aid in differentiating between OKCs, DCs, and RCs.
Shruthi et al. (2014) [89]	To understand the expression pattern of cytokeratins (CKs) 14 and 18 in dentigerous cysts, dental follicular tissue, adenomatoid odontogenic tumors (AOTs), and unicystic ameloblastomas.	Study Type: Immunohistochemical study. Sample Size: 20 dentigerous cysts, 20 reduced enamel epithelium/dental follicles, 10 follicular-type AOTs, 10 unicystic ameloblastomas (UCAs). Country/Region: India (Karnataka, Madhya Pradesh).	Immunohistochemistry using monoclonal antibodies for CK14 and CK18.	Histopathological examination using hematoxylin and eosin staining.	CK14 expression: Highest in AOT, moderate in dentigerous cysts and UCA, least in dental follicle/reduced enamel epithelium (REE). CK18: Negative in all cases. Significant differences in CK14 expression among the lesions, with AOT showing more percentage, positivity, and intensity compared to dentigerous cysts and UCA.	One-way ANOVA for mean percentage of CK14-positive cells (highest in AOT, least in DF/REE); T-test for percentage, positivity, intensity, and Remmele score (statistically significant differences between AOT and dentigerous cyst/UCA).	CK14 expression in AOT, dentigerous cyst, UCA, and DF/REE suggests a potential histogenetic relationship. The absence of CK18 in these lesions indicates its limited role in their pathogenesis. Further studies are needed to explore the oncofetal transformation and histogenesis of follicular-type AOT.
Swetha et al. (2014) [90]	To analyze the expression of inducible nitric oxide synthase (iNOS) in the epithelial lining of odontogenic keratocysts (OKCs), dentigerous cysts (DCs), and radicular cysts (RCs) to understand its role in the neoplastic nature and local aggressiveness of these cysts.	Study Type: Immunohistochemical study. Sample Size: 10 OKCs, 10 DCs, 10 RCs. Country/Region: India.	Immunohistochemistry using rabbit polyclonal antibody against iNOS.	Histopathological examination using hematoxylin and eosin staining.	iNOS expression observed in entire thickness of epithelial linings in 10 OKCs (40% intensely stained, 20% moderately stained, 20% mildly stained, 20% no staining). In DCs, 10% of cases showed intense staining, 20% moderate, 30% mild, 40% no staining. In RCs, 40% of cases showed mild staining, 60% no staining. Significant difference in iNOS staining intensity between OKCs, DCs, and RCs (*p* < 0.01).	ANOVA and Chi-square tests showed significant differences in iNOS expression between the cyst groups (*p* < 0.01).	iNOS overexpression in OKCs compared to DCs and RCs suggests a role in the aggressive behavior of OKCs, supporting the view that OKCs are neoplastic in nature.
Sudhakara et al. (2016) [91]	To analyze the expression of cytokeratin 14 (CK14) and vimentin in adenomatoid odontogenic tumor (AOT) and dentigerous cyst (DC) to understand their origin and pathogenesis.	Study Type: Retrospective immunohistochemical study. Sample Size: 16 AOTs (10 follicular, 6 extrafollicular), 15 DCs. Country/Region: India.	Immunohistochemistry using monoclonal antibodies for CK14 and vimentin.	Histopathological examination using hematoxylin and eosin staining.	CK14: Positive in 90% of follicular AOTs (FAOTs) and 100% of extrafollicular AOTs (EAOTs), positive in all DC cases. Vimentin: Positive in 44% of AOT cases, negative in 56%; in DC, 73% negative, 20% intermediate positive, 7% weak positive. CK14 predominantly expressed in Type B cells in AOT, suggesting odontogenic epithelial origin.	Descriptive statistics and measures of central tendency used to analyze results.	CK14 expression in AOT and DC supports their odontogenic epithelial origin. Vimentin expression in AOT is variable, suggesting a minor role in pathogenesis. The study indicates a potential origin from reduced enamel epithelium and dental lamina.
Saluja et al. (2019) [92]	To elucidate the role of cytokeratin-7 (CK7) in the pathogenesis of odontogenic cysts by immunohistochemistry.	Study Type: Immunohistochemical study. Sample Size: 15 dentigerous cysts (DCs), 12 odontogenic keratocysts (OKCs), 12 radicular cysts (RCs), and 8 control specimens (periampullary carcinoma, nasopalatine duct cysts, and dental follicle). Country/Region: India.	Immunohistochemistry using monoclonal mouse anti-human cytokeratin-7 antibody.	Histopathological examination using hematoxylin and eosin staining.	CK7 expression was highest in DCs (66.66%), followed by RCs (41.66%), and least in OKCs (16.6%). CK7 positive in suprabasal (60%) and superficial layers (40%) in DCs; in superficial and spinous layers in RCs and OKCs. Statistically significant difference in CK7 expression between DCs and OKCs (*p* = 0.009), but not between RCs and DCs or RCs and OKCs.	Chi-square test; significant differences with *p* ≤ 0.05.	CK7 expression correlates with the degree of epithelial differentiation. Well-differentiated epithelium (RC and DC) shows CK7 expression, while less well-differentiated epithelium (OKC) shows slight positivity. CK7 can be used to differentiate OKC from DC and RC.

### 4.6. Ki-67 and Other Proliferative Markers

Proliferative markers, particularly Ki-67, are instrumental in understanding the growth behavior, aggressiveness, and recurrence likelihood of odontogenic lesions such as odontogenic keratocysts (OKCs), dentigerous cysts (DCs), ameloblastomas (ABs), and unicystic ameloblastomas (UAs) [93,94]. Ki-67, a marker indicating cellular proliferation, shows notably higher levels in OKCs compared to DCs, suggesting a more aggressive growth pattern and a greater propensity for recurrence [56].

The interaction of Ki-67 with other markers like p63 and MCM3 provides deeper insights into the complex biology of these lesions, enabling clinicians to better predict their behavior and tailor treatment approaches accordingly. For instance, p63, which is associated with the regulation of epithelial cell proliferation and differentiation, shows higher expression in OKCs and ABs compared to DCs, correlating with their more aggressive behavior [95,96]. MCM3, another proliferation marker, also demonstrates higher expression in OKCs and ABs, further supporting their higher proliferative activity compared to DCs [97].

Elevated Ki-67 levels, particularly in OKCs and ABs, signal a higher risk of recurrence, guiding clinicians towards more aggressive management strategies, from surgical resections to closer post-operative monitoring. The incorporation of Ki-67 staining in routine diagnostic procedures has improved the stratification of recurrence risk, enabling clinicians to tailor follow-up intervals and treatment intensities based on individual patient profiles, thereby optimizing clinical outcomes [98,99]. For example, studies have shown that OKCs exhibit a significantly higher cellular proliferation index in the suprabasal layers compared to DCs, indicating their potential for more aggressive behavior and higher recurrence rates [56,94].

Additionally, research indicates that the expression of Ki-67 and MCM3 in different odontogenic lesions not only reflects their proliferative capacity but also aids in distinguishing between more and less aggressive types. For example, the mean Ki-67 labeling index in ameloblastomas is significantly higher than in DCs, highlighting the neoplastic nature of ameloblastomas compared to the more benign behavior of DCs [100]. Furthermore, the positive correlation between Ki-67 and p53 expression in OKCs and DCs underscores the role of these markers in understanding the pathogenesis and biological behavior of these lesions [101].

To modify and personalize therapy based on these markers, non-surgical approaches such as targeted therapies could be explored. For instance, lesions with high Ki-67 expression might benefit from treatments that inhibit cellular proliferation. The development of targeted inhibitors against specific pathways involved in cell proliferation, such as p63 or MCM3, could provide alternative or adjunctive treatments to traditional surgical methods. Additionally, personalized follow-up schedules based on Ki-67 levels could improve patient outcomes by ensuring timely intervention for recurrent lesions.

In summary, the evaluation of proliferative markers like Ki-67 marks a significant advancement in the field of oral health care. These markers provide crucial insights into how odontogenic lesions develop and respond to treatments, improving the prediction of outcomes and enabling more effective planning and execution of therapeutic strategies. Ongoing research into these markers is vital for refining management approaches and achieving better patient care outcomes [56,93,94,95,96,97,98,99,100,101] (Table 6).

**Table 6:** Proliferative markers (Ki-67, maspin, CD138, syndecan-1, p67, MCM-2, MCM-3, EGF, CD34, and COX-2).

Authors	Objective	Study Details	Marker Identification Method	Cyst/Tumor Diagnosis Method	Results	Statistical Estimates	Conclusion
Portes et al. (2020) [93]	To evaluate and compare the immunoexpression and immunostaining intensities of Ki-67 antigen in odontogenic keratocysts (OKCs) and dentigerous cysts (DCs) using computerized analysis.	Study Type: Immunohistochemical study. Sample Size: 15 OKCs, 6 DCs. Country/Region: Brazil.	Immunohistochemistry using monoclonal Ki-67 antibody (clone MIB-1) and computerized analysis with Aperio Technologies Inc. System.	Histopathological examination using hematoxylin and eosin staining.	There are no statistically significant differences in Ki-67 immunoexpression or staining intensities between OKCs and DCs. OKCs showed a significantly higher cellular proliferation index in suprabasal layers compared to basal layers. There are no significant differences in Ki-67 expression between OKCs from maxilla versus mandible or primary versus recurrent OKCs.	Mann–Whitney test, Student’s *t*-test, and Signal test used for statistical analysis; significance level set at α = 0.05.	Increased Ki-67 immunoexpression in the suprabasal layers of OKCs suggests a different biological behavior and more aggressive proliferation potential compared to DCs. Computerized evaluation provides a more reliable method for assessing immunoexpression.
Hammad et al. (2020) [94]	To investigate the expressions of maspin, syndecan-1, and Ki-67 in odontogenic keratocysts (OKCs) compared to dentigerous cysts (DCs) and ameloblastomas (ABs).	Study Type: Immunohistochemical analysis. Sample Size: 26 OKCs, 11 DCs, 10 ABs. Country/Region: Jordan.	Immunohistochemistry using antibodies against maspin, syndecan-1, and Ki-67.	Histopathological examination using hematoxylin and eosin staining.	Maspin: Lower expression in OKC and DC compared to AB, but not statistically significant. Syndecan-1: Lower expression in OKC and AB compared to DC, but not statistically significant. Ki-67: Significantly higher expression in OKC compared to DC (*p* < 0.05), like AB.	ANOVA and Student’s *t*-test used; significant differences in Ki-67 scores between OKC and DC (*p* < 0.05).	Ki-67 expression indicates higher proliferative activity in OKC similar to AB, higher than in DC. Expressions of maspin and syndecan-1 are not significantly different among OKC, AB, and DC, suggesting further investigation into the biological behavior of OKC is needed.
Alsaegh et al. (2020) [95]	To investigate p63 immunoexpression and its relation to the proliferation of epithelial lining in dentigerous cyst (DC), odontogenic keratocyst (OKC), and ameloblastoma (AB).	Study Type: Immunohistochemical study. Sample Size: 12 DCs, 9 OKCs, 15 ABs. Age Range: Mean age: 40.11 years (±17.567). Country/Region: United Arab Emirates, China.	Immunohistochemistry using antibodies against p63 and Ki-67.	Histopathological examination using hematoxylin and eosin staining.	p63 expression: Significant difference among DCs, OKCs, and ABs (*p* = 0.048). Higher in OKCs compared to DCs (*p* = 0.018). Ki-67: Significant difference among DCs, OKCs, and ABs (*p* = 0.022). Higher in OKCs compared to DCs (*p* = 0.007). Positive correlation between p63 and Ki-67 in DCs (σ = 0.757, *p* = 0.004) and OKCs (σ = 0.741, *p* = 0.022). No correlation in AB group.	Kruskal–Wallis test, Mann–Whitney U test, Spearman’s correlation analysis (*p* < 0.05).	The diverse expression and correlation of p63 with proliferation in odontogenic lesions suggest different roles and pathways of ΔNp63 in odontogenic tumors versus cysts, aiding in understanding their pathogenesis and behavior.
Jaafari-Ashkavandi et al. (2015) [96]	To investigate the diagnostic impact of P63 protein on dentigerous cysts and various types of ameloblastoma and compare its expression with the Ki-67 proliferation marker.	Study Type: Cross-sectional retrospective immunohistochemical study. Sample Size: 25 dentigerous cysts, 21 unicystic ameloblastomas, 17 conventional ameloblastomas. Age Range: Mean age: 27 ± 15.2 years. Country/Region: Iran.	Immunohistochemistry using monoclonal anti-P63 antibody and Ki-67 antibody.	Histopathological examination using hematoxylin and eosin staining.	P63 expression higher in ameloblastoma compared to unicystic ameloblastoma and dentigerous cysts (*p* < 0.05). No significant difference in P63 expression between unicystic ameloblastoma and dentigerous cysts. A 90% cut-off point for basal layer gave 88% sensitivity and 78% specificity to distinguish more invasive lesions. No correlation between P63 and Ki-67 immunostaining in the three study groups.	Mann–Whitney test, T-test, correlation coefficient test, ROC curve analysis; significant differences with *p* < 0.05.	Higher P63 expression indicates more aggressive odontogenic lesions. No correlation found between P63 and Ki-67, suggesting different roles in tumor genesis and proliferation. P63 could be a useful diagnostic marker for aggressive odontogenic lesions.
Jaafari-Ashkavandi et al. (2019) [97]	To investigate the proliferative activity of dentigerous cysts (DCs), odontogenic keratocysts (OKCs), and ameloblastomas (ABs) using minichromosome maintenance 3 (MCM3) and Ki-67 proliferation markers.	Study Type: Cross-sectional immunohistochemical study. Sample Size: 11 DCs, 14 OKCs, 15 ABs (11 solid, 4 unicystic). Age Range: Mean age: 28.9 ± 18.6 years. Country/Region: Iran.	Immunohistochemistry using anti-MCM3 and anti-Ki-67 antibodies.	Histopathological examination using hematoxylin and eosin staining.	MCM3 and Ki-67 are expressed in all cases. Higher MCM3 and Ki-67 expression in OKCs and ABs compared to DCs. MCM3 expression higher than Ki-67 in all groups. Expression more prominent in basal and parabasal layers of OKCs, peripheral cells of ameloblastomas, and basal layer of DCs. There is no significant difference in expression between inflamed and non-inflamed cysts.	ANOVA and Tukey tests showed significant differences in MCM3 and Ki-67 expression among all groups (*p* < 0.000). Spearman’s correlation test showed weak positive correlation between MCM3 and Ki-67 (ρ = 0.57, *p* = 0.002).	MCM3 is a more sensitive proliferation marker than Ki-67. Higher expression of both markers in OKCs and ABs suggests higher proliferative activity and supports their aggressive behavior. MCM3 and Ki-67 are reliable markers for assessing proliferation in odontogenic lesions.
Brito-Mendoza et al. (2018) [98]	To compare the expression of Ki-67, syndecan-1 (CD138), and the molecular triad RANK, RANKL, and OPG in odontogenic keratocysts (OKCs), unicystic ameloblastomas (UAs), and dentigerous cysts (DCs).	Study Type: Immunohistochemical study. Sample Size: 22 OKCs, 19 UAs, 17 DCs. Country/Region: Mexico, Uruguay, Brazil.	Immunohistochemistry using antibodies for Ki-67, CD138, RANK, RANKL, and OPG.	Histopathological examination using hematoxylin and eosin staining.	Higher Ki-67 expression in OKC compared to UA and DC (*p* < 0.0001). Greater loss of CD138 in UA compared to OKC (*p* = 0.0034). Higher RANKL expression in UA epithelium and stroma (*p* = 0.0002, *p* = 0.0004). DC showed lower expression of all markers.	Chi-square test, Kruskal–Wallis test, Tukey–Kramer method, Spearman’s Rho; significant differences with *p* < 0.05.	Increased RANKL expression and reduced CD138 expression in UA indicate higher invasive and destructive potential. The higher proliferation rate in OKC is related to its continuous intrabony growth. DC expansion does not seem to be related to these factors.
Modi et al. (2018) [99]	To compare the expression of Ki-67 in odontogenic keratocysts (OKCs), radicular cysts (RCs), and dentigerous cysts (DCs) to understand their proliferative activity and aggressive behavior.	Study Type: Immunohistochemical study. Sample Size: 15 OKCs, 15 RCs, 15 DCs. Country/Region: India (Gujarat and Uttar Pradesh).	Immunohistochemistry using Ki-67 monoclonal antibody (streptavidin–biotin detection system HRP-DAB).	Histopathological examination using hematoxylin and eosin staining.	Ki-67 positive cells were highest in OKC epithelium compared to DC and RC. Ki-67 labeling index (LI) in OKC: 12.76 ± 4.78; DC: 5.87 ± 4.24; RC: 5.08 ± 3.11. Ki-67 expression significantly higher in suprabasal layer of OKC (19.66 ± 7.89) compared to DC (3.60 ± 2.31) and RC (3.12 ± 2.19). No significant difference in Ki-67 LI in the basal layer among all groups.	Statistically significant differences in Ki-67 expression in suprabasal layers between OKC and DC (*p* < 0.0001), and between OKC and RC (*p* < 0.0001). No significant difference between DC and RC (*p* = 0.558).	Increased Ki-67 expression in the suprabasal cell layers of OKC indicates higher proliferative activity and aggressive behavior, suggesting that OKC may be neoplastic rather than a developmental cyst.
Alsaegh et al. (2017) [102]	To evaluate COX-2 expression and its correlation with the proliferation of odontogenic epithelium in dentigerous cysts (DCs) and ameloblastomas (ABs).	Study Type: Immunohistochemical study. Sample Size: 16 DCs, 17 ABs. Age Range: 12–74 years; mean age: 36.2 years. Country/Region: China, UAE, Iraq, Japan.	Immunohistochemistry using antibodies for COX-2 and Ki-67.	Histopathological examination using hematoxylin and eosin staining.	Ki-67 expression: DCs (absent: 25%; weak: 50%; mild: 12.5%; strong: 12.5%); ABs (absent: 23.52%; weak: 41.17%; mild: 17.64%; strong: 17.64%). COX-2 expression: DCs (absent: 18.75%; weak: 18.75%; mild: 43.75%; strong: 18.75%); ABs (absent: 5.89%; weak: 29.41%; mild: 52.94%; strong: 11.76%). Significant positive correlation between Ki-67 and COX-2 expression in DCs (*p* = 0.018) and ABs (*p* = 0.004).	Mann–Whitney U test and Spearman’s rank correlation coefficient; significant differences with *p* < 0.05.	COX-2 and Ki-67 expression indicate higher proliferative activity in odontogenic epithelium of DCs and ABs, suggesting COX-2 as a potential target in managing these lesions.
Güler et al. (2012) [103]	To investigate the association between inflammation and the expression of cell cycle markers Ki-67 and MCM-2 in dental follicles (DF) and odontogenic cysts.	Study Type: Immunohistochemical study. Sample Size: 70 dental follicles (DF) and 20 odontogenic cysts (6 radicular cysts (RCs), 7 dentigerous cysts (DCs), 7 keratocystic odontogenic tumors (KCOTs)). Age Range: DFs: mean age 27 years (range 16–68 years); Cysts: mean age 39 years (range 22–67 years). Country/Region: Turkey.	Immunohistochemistry using Ki-67 and MCM-2 antibodies.	Histopathological examination using hematoxylin and eosin staining.	Ki-67 expression: DF (9.64 ± 5.99), RC (12.17 ± 4.49), DC (7.43 ± 3.99), KCOT (16 ± 13.46) MCM-2 expression: DF (6.34 ± 3.81), RC (19.17 ± 3.76), DC (7 ± 4.25), KCOT (15.43 ± 14.04). Significant correlation between inflammation and Ki-67/MCM-2 expressions in DFs and cysts (*p* < 0.01).	Kruskal–Wallis test, Mann–Whitney U test, Student’s *t*-test; significant differences with *p* < 0.05.	Higher MCM-2 expression in RCs compared to KCOTs suggests a potential sensitivity to inflammation. Both Ki-67 and MCM-2 are useful in assessing proliferative activity and the influence of inflammation in odontogenic cysts and dental follicles.
Kim et al. (2003) [104]	To evaluate the comparative proliferative activity and apoptosis in odontogenic keratocysts (OKCs) associated with or without an impacted tooth and between unilocular and multilocular OKC varieties.	Study Type: Immunohistochemical study. Sample Size: 32 OKCs (16 with impacted tooth, 16 without impacted tooth), 10 dentigerous cysts (DCs).	Immunohistochemistry using Ki-67 for proliferation and TUNEL method for apoptosis.	Histopathological examination using hematoxylin and eosin staining.	OKCs showed greater proliferative potential and more apoptotic reactions than DCs. Proliferating cells primarily in the suprabasal layer and apoptotic cells in the superficial layer of OKCs. There is no significant difference in proliferative activity or apoptosis between OKCs associated with or without impacted teeth, or between unilocular and multilocular OKC varieties.	Statistical significance not explicitly stated.	OKCs are characterized by increased cell proliferation and apoptosis, indicating a unique proliferative and differentiation process. The aggressive behavior or recurrence in multilocular OKCs is likely due to incomplete removal or other contributing factors rather than intrinsic growth or apoptosis.
de Oliveira et al. (2011) [105]	To evaluate the biological profile of odontogenic epithelium by immunolabeling of epidermal growth factor receptor (EGFR), Ki-67, and survivin in keratocystic odontogenic tumors (KOTs), dentigerous cysts (DCs), and pericoronal follicles (PFs).	Study Type: Immunohistochemical study. Sample Size: 13 KOTs, 14 DCs, 9 PFs. Country/Region: Brazil.	Immunohistochemistry using antibodies for EGFR, Ki-67, and survivin.	Histopathological examination using hematoxylin and eosin staining.	KOTs showed the highest proliferation rate among the three groups, mainly in suprabasal layers. EGFR immunolabeling observed mainly in the cytoplasm in basal and suprabasal layers of KOTs, and in the suprabasal layer of DCs. Survivin immunolabeling showed a greater percentage of positive cells in the suprabasal layer of KOTs. PFs showed the highest percentage of survivin-positive cells, with immunolabeling in both the membrane and cytoplasm.	Chi-square test, Fisher’s exact test, Spearman coefficient, Mann–Whitney test, Wilcoxon test; significant differences with *p* < 0.01.	Epithelial cells in KOTs demonstrate stimulus-independent proliferative characteristics, suggesting a suprabasal proliferative compartment maintained by inhibition of apoptosis. In DCs, the basal layer proliferates in response to stimuli. PFs, though showing low proliferative activity, have a high capacity to respond to stimuli, indicating potential for odontogenic lesion development.
Nadalin et al. (2011) [106]	To assess the immunohistochemical expression of syndecan-1 (CD138) and Ki-67 in radicular cysts (RCs), dentigerous cysts (DCs), and keratocystic odontogenic tumors (KOTs).	Study Type: Immunohistochemical study. Sample Size: 35 RCs, 22 DCs, 17 KOTs. Age Range: RC: Mean age 42.2 years; DC: Mean age 29 years; KOT: Mean age 45.8 years. Country/Region: Brazil.	Immunohistochemistry using antibodies for syndecan-1 (CD138) and Ki-67.	Histopathological examination using hematoxylin and eosin staining.	Syndecan-1: High expression in 85.7% RCs, 95.5% DCs, and 94.1% KOTs. Ki-67: Higher suprabasal expression in KOTs compared to RCs and DCs (*p* < 0.0001). Positive correlation between syndecan-1 and Ki-67 in RCs (*p* = 0.01) and KOTs (*p* = 0.01). Intense inflammation reduces syndecan-1 expression in RCs and KOTs.	Fisher’s exact test, Spearman’s correlation coefficient; significant differences with *p* < 0.05.	Syndecan-1 is not a determinant factor in the distinct histopathological features and biological behavior of the studied lesions. However, a positive correlation between syndecan-1 and Ki-67 indicates its potential role in cell proliferation in RCs and KOTs.
Naruse et al. (2017) [107]	To identify the most useful markers for predicting the recurrence of keratocystic odontogenic tumors (KCOTs) by evaluating the expression profiles of Ki-67, CD34, and podoplanin.	Study Type: Retrospective immunohistochemical study. Sample Size: 65 tumor samples from 63 patients. Age Range: Median age: 41 years (range 10–87 years). Country/Region: Japan.	Immunohistochemistry using antibodies for Ki-67, CD34, and podoplanin.	Histopathological examination using hematoxylin and eosin staining.	High Ki-67, CD34, and podoplanin expression levels were associated with tumor recurrence. Univariate analysis revealed a significant association between high CD34 expression and tumor recurrence (*p* = 0.034), as well as conservative surgical treatment (*p* = 0.003). Multivariate analysis identified conservative treatment as the greatest independent risk factor for tumor recurrence (odds ratio = 13.337, *p* = 0.018).	Univariate and multivariate logistic regression analyses; significant differences with *p* < 0.05.	Overexpression of CD34 may be a potent predictor of tumor recurrence. Radical treatment of teeth in contact with tumors is recommended to prevent recurrence. Conservative treatment was significantly associated with higher recurrence rates.
Selvi et al. (2012) [108]	To investigate the role of Ki-67 and argyrophilic nucleolar organizing regions (AgNORs) in differentiating recurrent and non-recurrent keratocystic odontogenic tumors (KCOTs) and to compare the correlation between these markers.	Study Type: Retrospective immunohistochemical study. Sample Size: 22 KCOT cases. Country/Region: Turkey.	Immunohistochemistry for Ki-67 and silver staining for AgNOR.	Histopathological examination using hematoxylin and eosin staining.	Recurrence in three patients (13.6%) during a mean follow-up period of 37.8 months (about 3 years). Significantly higher Ki-67 and AgNOR counts in recurrent lesions compared to non-recurrent lesions (*p* = 0.045 for Ki-67; *p* = 0.049 for AgNOR). Positive correlation between Ki-67 and AgNOR counts (r = 0.853, *p* = 0.0001).	Mann–Whitney U test, Fisher’s exact test, and Spearman’s correlation; significant differences with *p* < 0.05.	Ki-67 and AgNOR may serve as prognostic markers for KCOT recurrence. The findings support the classification of KCOT as an odontogenic tumor and suggest that enucleation with curettage or decompression followed by enucleation with curettage is effective despite a relatively high recurrence rate. Conservative treatment should be chosen carefully, avoiding cases with coronoid invasion, cortical lysis, or tissue invasion.
Ba et al. (2010) [109]	To investigate the relationship between radiographic appearance and epithelial cell proliferation in keratocystic odontogenic tumors (KCOTs).	Study Type: Retrospective radiographic and immunohistochemical study. Sample Size: 284 KCOT cases for radiographic analysis; 30 cases for Ki-67 immunohistochemical analysis. Age Range: Mean age: 32 years (range 9–87 years). Country/Region: China and Japan.	Immunohistochemistry using Ki-67 antibody.	Histopathological examination using hematoxylin and eosin staining.	Radiographic types: Unilocular (64.79%), multilocular (21.90%), multiple (9.79%), NBCCS-associated (3.52%). Ki-67 expression: Higher in NBCCS-associated KCOTs compared to solitary and multiple KCOTs (*p* = 0.018, 0.002). Significant difference in Ki-67 expression between multilocular and unilocular/NBCCS-associated KCOTs (*p* = 0.000). No significant difference between solitary and multiple KCOTs (*p* = 0.220).	ANOVA, least significant difference test; significant differences with *p* < 0.05.	A high correlation exists between the biological behavior of KCOTs and their imaging features. The solitary KCOTs seem less biologically aggressive and should be classified as cysts rather than tumors. More than half of KCOTs manifest as ordinary cysts.
Mendes et al. (2011) [110]	To investigate the association between the expression of cyclooxygenase-2 (COX-2) in keratocystic odontogenic tumors (KCOTs) and more commonly used markers, such as p53 and Ki-67.	Study Type: Immunohistochemical study. Sample Size: 20 KCOT biopsy specimens.	Immunohistochemistry using anti-COX-2, anti-Ki-67, and anti-p53 monoclonal antibodies.	Histopathological examination using hematoxylin and eosin staining.	COX-2 expression: Mild to strong in 100% of cases. p53 expression: Positive in 75% of cases. Ki-67 expression: Positive in 90% of cases. No statistically significant difference between the expressions of COX-2, Ki-67, and p53.	Statistical relevance of differences between COX-2, Ki-67, and p53 expressions not found.	COX-2 may be an important marker involved in the biological behavior of KCOTs. Despite its rare usage in assessing KCOTs, its role in tumorigenesis suggests its significance. Larger studies are required to understand the possible role of COX-2 in KCOT pathogenic mechanisms.
Nafarzadeh et al. (2013) [100]	To investigate the expression of PCNA and Ki-67 in dental follicle, dentigerous cyst, unicystic ameloblastoma, and ameloblastoma to assess their proliferative status.	Study Type: Immunohistochemical study. Sample Size: 60 samples: 15 each of dental follicle, dentigerous cyst, unicystic ameloblastoma, and ameloblastoma. Country/Region: Iran.	Immunohistochemistry using anti-Ki-67 and anti-PCNA monoclonal antibodies.	Histopathological examination using hematoxylin and eosin staining.	Ki-67: Weak in dental follicle (12), moderate in dentigerous cyst (14), intense in ameloblastoma (10). PCNA: Weak in dental follicle (12), moderate in dentigerous cyst (14), intense in ameloblastoma (13). Correlation coefficient between Ki-67 and PCNA: 0.88, statistically significant (*p* < 0.001).	Chi-square test, Pearson correlation, one-way ANOVA; significant differences with *p* < 0.05.	Significant differences in Ki-67 and PCNA expressions among dental follicle, dentigerous cyst, unicystic ameloblastoma, and ameloblastoma. Ki-67 and PCNA can be used to estimate proliferative status and aggressiveness, aiding in understanding the biological behavior and prognosis of these lesions.
Kuroyanagi et al. (2009) [111]	To determine prognostic factors for the recurrence of keratocystic odontogenic tumors (KCOTs) following simple enucleation by examining clinicopathologic and immunohistochemical findings.	Study Type: Retrospective study. Sample Size: 32 subjects diagnosed with KCOT. Country/Region: Japan.	Immunohistochemistry using antibodies for Ki-67 and p53.	Histopathological examination using hematoxylin and eosin staining.	Recurrence rate: 12.5% (4 out of 32 subjects). High Ki-67 expression (>10% LI) in basal layer: 75.0% in recurrent group vs. 14.3% in non-recurrent group (*p* = 0.025). p53 expression: 75.0% in recurrent group vs. 39.3% in non-recurrent group (*p* = 0.295). Hazard risk for recurrence with high Ki-67 expression: 4.02 (95% CI 1.42–18.14, *p* = 0.009).	Cox proportional hazard model, *p* < 0.05.	High Ki-67 expression in the basal layer of KCOTs is significantly associated with recurrence. Evaluating Ki-67 expression at diagnosis may help guide the use of appropriate adjunctive surgical procedures to prevent recurrence and serve as a prognostic marker.
Ono et al. (2022) [112]	To determine the clinical, pathological, and genetic characteristics of multiple orthokeratinized odontogenic cysts (OOCs).	Study Type: Clinical, pathological, and genetic study. Sample Size: 3 cases of multiple OOCs (total of 7 OOCs). Age Range: Mean age: 25.3 years (range 18–38 years). Country/Region: Japan.	Immunohistochemistry using antibodies for Ki-67 and Bcl-2; next-generation sequencing (NGS) for PTCH1 mutations.	Histopathological examination using hematoxylin and eosin staining.	Clinical findings: All OOCs were in posterior regions; 57.1% associated with impacted teeth; no recurrence observed. Histological findings: Cysts lined by orthokeratinized stratified squamous epithelium; no OKC features observed. Immunohistochemical findings: Low Ki-67 labeling index (mean 9.43%); no Bcl-2 expression. Genetic findings: No pathogenic PTCH1 mutations detected.	No specific statistical tests mentioned.	Multiple OOCs occur more often in younger patients and show mild biological behavior with no recurrence, distinguishing them from OKCs. Both multiple and solitary OOCs are considered related diseases within the entity of odontogenic cysts and are distinct from OKCs.
Park et al. (2020) [113]	To assess changes in histology and expression of proliferation markers in odontogenic keratocysts (OKCs) before and after decompression treatment.	Study Type: Clinical and immunohistochemical study. Sample Size: 38 OKC tissue samples from 19 patients. Age Range: Mean age: 38.8 years (range 19–81 years). Country/Region: South Korea.	Immunohistochemistry using antibodies for Bcl-2, EGFR, Ki-67, P53, PCNA, and SMO.	Histopathological examination using hematoxylin and eosin staining.	Decompression period: 4 to 12 months (mean 7.3 months). No significant change in Bcl-2, Ki-67, P53, PCNA, and SMO values before and after decompression. Significant change in EGFR values before and after decompression (*p* = 0.040). There is no correlation between clinical shrinkage and morphologic changes or expression of proliferation and growth markers. OKCs recurred in 3 patients’ post-decompression.	Paired *t*-test, Wilcoxon signed ranks test; *p* < 0.05 considered significant.	Decompression does not significantly change the biological behavior or recurrence rate of OKCs. EGFR values changed significantly, but no other markers showed significant change, indicating decompression does not reduce the aggressive behavior of OKCs.
Coşarcă et al. (2016) [101]	To analyze the immunoexpression of Ki67, p53, MCM3, and PCNA in dental follicles of impacted teeth, dentigerous cysts (DCs), and keratocystic odontogenic tumors (KCOTs) to evaluate their proliferative capacity and evolutionary behavior.	Study Type: Immunohistochemical study. Sample Size: 62 dental follicles of impacted teeth, 20 DCs, 20 KCOTs. Country/Region: Romania.	Immunohistochemistry using antibodies for Ki67, p53, MCM3, and PCNA.	Histopathological examination using hematoxylin and eosin staining.	Dental follicles: Positive for PCNA (96.77%), Ki67 (90.32%), MCM3 (74.19%), and p53 (64.51%). Significant differences in Ki67, p53, and MCM3 between basal and parabasal layers in DCs and KCOTs. Positive correlation between Ki67 and MCM3 in basal and parabasal layers of KCOTs. Ki67 and MCM3 are useful in distinguishing between DCs and KCOTs.	Chi-square test, Mann–Whitney test, Spearman’s correlation; significant differences with *p* < 0.05.	Ki67 and MCM3 are the most useful markers for evaluating the proliferative capacity and distinguishing between DCs and KCOTs. KCOTs show more aggressive behavior and higher proliferative capacity compared to DCs.
Jabbarzadeh et al. (2021) [114]	To assess the Ki-67 labeling index (LI) in odontogenic cysts and tumors through a systematic review and meta-analysis.	Study Type: Systematic review and meta-analysis. Sample Size: 608 lesions.	Immunohistochemistry for Ki-67.	Histopathological examination using hematoxylin and eosin staining.	Ki-67 LI in benign odontogenic tumors: <5%. Ki-67 LI in malignant odontogenic tumors: >15.3%. Highest Ki-67 LI in ameloblastoma (4.39 ± 0.47) among benign tumors. Highest Ki-67 LI in odontogenic cysts: odontogenic keratocyst (OKC) (3.58 ± 0.51). Significant difference in Ki-67 LI between malignant and benign odontogenic lesions (*p* < 0.001).	Random-effects model for pooled LI mean at 95% CI; significant heterogeneity (Q = 743.03, df = 28, *p* < 0.001, I^2^ = 96.23).	Ki-67 LI is a reliable marker for distinguishing between benign and malignant odontogenic lesions. The high Ki-67 LI in OKCs suggests they are more like tumors, implying a need for tumor-like treatment plans.
Bhola et al. (2024) [115]	To compare the expression of MCM-3 and Ki-67 in odontogenic cysts and evaluate the sensitivity of these markers to inflammation.	Study Type: Immunohistochemical study. Sample Size: 37 dentigerous cysts, 37 odontogenic keratocysts (OKCs), 27 radicular cysts. Country/Region: Romania.	Immunohistochemistry using antibodies for Ki-67 and MCM-3.	Histopathological examination using hematoxylin and eosin staining.	Higher labeling index (LI) of MCM-3 compared to Ki-67 in all study groups. Positive correlation of Ki-67 LI with inflammation. MCM-3 proteins proved more accurate for determining proliferation potential and were not sensitive to external stimuli like inflammation, unlike Ki-67.	Statistical analysis with *p* < 0.05 considered significant.	MCM-3 is a more accurate marker for determining proliferation potential and is not influenced by inflammation, unlike Ki-67, making it more reliable for evaluating odontogenic cysts.
Embaló et al. (2018) [116]	To evaluate the metabolism and epithelial cell proliferation of odontogenic keratocyst (OKC), dentigerous cyst (DC), and unicystic ameloblastoma (UA) by quantifying the nucleolar organizing regions (AgNORs) and Ki-67 protein immunoexpression.	Study Type: Retrospective study. Sample Size: 16 OKCs, 16 DCs, 16 UAs.	Immunohistochemistry for Ki-67 and AgNOR.	Histopathological examination using hematoxylin and eosin staining.	Ki-67 and AgNOR counts were significantly higher in OKC compared to DC and UA (*p* < 0.001). Ki-67-positive cells were higher in suprabasal cell layers of OKC with uniform distribution, while a few were observed predominantly in basal cell layer in DC and UA. AgNOR count was significantly higher in the OKC basal cell layers and observed throughout the lining epithelium of DC and UA. OKC presented high metabolism and cellular proliferation compared to DC and UA, suggesting its aggressive clinical behavior and high recurrence rate.	Significant differences with *p* < 0.001.	Ki-67 and AgNOR reinforce the aggressive character of OKC, presenting high metabolism and cellular proliferation compared to DC and UA, possibly due to its more aggressive clinical behavior and high recurrence rate.
Kucukkolbasi et al. (2014) [117]	To assess the cell proliferation activity of dental follicles (DFs) surrounding asymptomatic impacted third molar teeth using the Ki-67 proliferation marker and to evaluate the variation of cell proliferation depending on age.	Study Type: Immunohistochemical study. Sample Size: 44 specimens of DFs. Age Range: 18 to 62 years (mean age: 32 years). Country/Region: Turkey.	Immunohistochemistry using Ki-67 monoclonal antibody.	Histopathological examination using hematoxylin and eosin staining.	Ki-67 expression: 60% in Group 1 (18–29 years), 75% in Group 2 (30+ years). Significant differences in Ki-67 expression between the two age groups in both basal and suprabasal layers. Histological examination showed higher squamous proliferation and inflammation in older patients. Squamous metaplasia was observed in all follicles, indicating a potential early sign of developing odontogenic lesions.	Statistically significant differences with *p* < 0.05.	DFs have more proliferative potential in older individuals compared to younger ones. Squamous metaplasia may be an early sign of developing odontogenic lesions, and histopathological changes could be found in DFs without clinical and radiographic alterations.
Cimadon et al. (2014) [118]	To evaluate the proliferative potential and cell proliferation rate of odontogenic epithelial cells using AgNOR and Ki-67, and to perform immunohistochemical staining for EGFR.	Study Type: Immunohistochemical study. Sample Size: 42 cases of pericoronal follicles of impacted third molars.	Silver impregnation technique for AgNOR, immunohistochemical staining for Ki-67 and EGFR.	Histopathological examination using hematoxylin and eosin staining.	Mean AgNORs per nucleus (mAgNOR): 1.43 (range: 1.0–2.42), with significant differences among pericoronal follicles from upper and lower teeth (*p* = 0.041). Ki-67 immunostaining was negative in all cases. EGFR immunolabeling was mainly cytoplasmic and more intense in islands and cords compared to the reduced epithelium of the enamel organ.	Significant differences with *p* < 0.05.	Odontogenic epithelial cells in some pericoronal follicles have proliferative potential, suggesting their association with the development of odontogenic lesions. Non-erupted teeth, especially of the lower jaw, should be monitored and possibly removed.
Chaturvedi et al. (2022) [119]	To distinguish aggressive from nonaggressive benign odontogenic tumors using the immunohistochemical expression of Ki-67 and Glypican-3 (GPC3).	Study Type: Immunohistochemical study. Sample Size: 20 solid ameloblastomas (8 follicular, 8 plexiform, 4 acanthomatous), 4 unicystic ameloblastomas, 28 keratocystic odontogenic tumors (KCOTs), 5 adenomatoid odontogenic tumors, and 2 calcifying cystic odontogenic tumors. Country/Region: India.	Immunohistochemistry using antibodies for Ki-67 and GPC3.	Histopathological examination using hematoxylin and eosin staining.	Ki-67: Positive correlation with aggressiveness (*p* < 0.001). GPC3: More useful than Ki-67 in distinguishing aggressiveness among aggressive tumors (*p* < 0.001). Intensity of GPC3: Maximum in plexiform ameloblastoma, followed by follicular and acanthomatous ameloblastomas, and KCOTs. GPC3 is not expressed in unicystic ameloblastomas.	Pearson correlation coefficient; *p* < 0.001 considered highly significant.	Ki-67 and GPC3 are valuable markers for differentiating aggressive from nonaggressive benign odontogenic tumors. GPC3 is more sensitive in determining the aggressiveness among aggressive odontogenic tumors.
Yamasaki et al. (2024) [120]	To describe the transformation of an odontogenic keratocyst (OKC) into a solid variant of odontogenic keratocyst/keratoameloblastoma (SOKC/KA) during long-term follow-up and analyze genetic mutations.	Study Type: Case report and genetic analysis. Sample Size: Single case study. Age Range: 26-year-old man at initial presentation. Country/Region: Japan.	Immunohistochemistry (IHC) for p53, Ki-67, BRAF, calretinin, β-catenin, CD56, p-S6, p-ERK1/2; Genetic panel sequencing for mutations.	Histopathological examination using hematoxylin and eosin staining; CT imaging.	Initial diagnosis: OKC. Recurrence: transformed to SOKC/KA with higher Ki-67 (~10%) and p53 positivity compared to primary lesion. Genetic mutations: APC (p.Arg876*), KRAS (p.Gly13Asp), and TP53 (p.Val31Ile). IHC: p-S6 and p-ERK1/2 positive in recurrent lesions.	Not applicable.	OKC can transform into SOKC/KA upon recurrence, indicated by increased proliferative activity and genetic mutations. The study suggests a close histogenetic relationship between OKC and SOKC/KA, and emphasizes the importance of genetic analysis in understanding tumor behavior.
Dong et al. (2010) [121]	To analyze the clinicopathologic features of 61 cases of orthokeratinized odontogenic cysts (OOCs) in a Chinese population.	Study Type: Clinicopathologic analysis and immunohistochemical study. Sample Size: 61 cases of OOC. Age Range: 13 to 75 years (average 38.93 years). Country/Region: China.	Immunohistochemical expression of Ki-67 and p63.	Clinicopathologic features and radiographic records.	90.16% of cases are mandible, 9.84% are maxilla. 87.04% unilocular radiolucencies, 12.96% multilocular. 50% associated with an impacted tooth. No recurrence in 42 patients after 76.8 months (about 6 and a half years) follow-up	Expression levels of Ki-67 and p63 were significantly lower in OOCs compared to KCOTs (*p* < 0.001).	OOC is clinicopathologically distinct from KCOT and should constitute its own clinical entity.
Mustansir-Ul-Hassnain et al. (2021) [122]	1. Analysis of histopathologic findings in odontogenic cysts before and after decompression. 2. Analysis of Ki-67 expression in odontogenic jaw cysts before and after decompression.	Study Type: Original Research Article. Sample Size: 10 cases of odontogenic cysts. Age Range: Average age 26 ± 9.2 years (range not specified). Country/Region: Greater Noida, Uttar Pradesh, India.	Immunohistochemistry using Ki-67 monoclonal antibody.	Histopathologic examination of incisional and excisional biopsies before and after decompression.	Fewer Ki-67 + cells in radicular cysts, dentigerous cysts, and sialo-odontogenic cyst compared to odontogenic keratocysts (OKCs). Average scores were 2.2 before and 1 after decompression (statistically significant difference).	Two-tailed *p* value < 0.0001. Confidence interval 95%. Hazard ratio for recurrence not applicable.	The proliferative activity, evaluated by Ki-67 marker, was significantly greater in the epithelial lining before decompression compared to after decompression. Decompression significantly diminishes the proliferative rate of the cystic epithelial lining.
Trujillo-González et al. (2022) [123]	To evaluate the histological effects of decompression treatment on OKC, including cell proliferation and apoptosis of epithelial cyst.	Study Type: Original Study. Sample Size: 21 samples. Age Range: 9–58 years. Country/Region: Venezuela, Uruguay.	Immunohistochemistry (Ki-67, MCM4/7, Bax, Bcl2).	Surgical decompression with histological evaluation and immunohistochemical staining.	Increased inflammation (*p* = 0.029), loss of parakeratinization (*p* = 0.007), absence of palisade cell distribution (*p* = 0.002). No significant changes in expression of Ki-67 (*p* = 0.323), MCM4/7, Bax, or Bcl-2.	*p* values: 0.029 (inflammation), 0.007 (parakeratinization), 0.002 (palisade cell distribution), 0.323 (Ki-67), 0.079 (MCM4/7), 0.392 (Bax), Bcl-2 not specified.	Surgical decompression induces structural changes in OKC but does not significantly alter cell proliferation or apoptosis.
Zhou et al. (2022) [124]	To demonstrate the clinicopathological and radiological features of orthokeratinized odontogenic cysts (OOCs) and analyze the epithelial cell proliferative activity between OOCs and odontogenic keratocysts (OKCs).	Study Type: retrospective clinicopathological and radiological analysis. Sample Size: 48 OOC cases and 20 OKC cases. Age Range: 13 to 61 years. Country/Region: Shanghai Ninth People’s Hospital.	Immunohistochemistry was performed using a DAKO AutostainerLink 48, with paraffin-embedded samples cut into 4-μm sections, deparaffinized, and rehydrated. Ki-67 and cyclin D1 antibodies were used for staining.	Histological examination and radiological imaging (panoramic radiographs and CT) were used to diagnose OOCs. The presence of orthokeratinized stratified squamous epithelial lining characterized OOCs.	Demographic Findings: 28 males and 20 females, with an average age of 33.50 years. Location: 40 cases in the mandible and 8 in the maxilla. Radiological Features: All OOCs were unilocular radiolucencies with well-defined margins, 83.33% showed buccolingual expansion. Histological Features: Thin, uniform orthokeratinized lining epithelium with a prominent granular cell layer. Proliferative Activity: Ki-67 and cyclin D1 expression were significantly lower in OOCs compared to OKCs (*p* < 0.001). Treatment: 40 cases treated with enucleation, 8 with decompression followed by enucleation. Follow-Up: Average follow-up of 32.50 ± 27.58 months (about 2 and a half years), with a 4.44% recurrence rate.	Ki-67 Expression: OOCs: 2.50% ± 0.25%, OKCs: 12.50% ± 1.42%, *p* < 0.001. Cyclin D1 Expression: OOCs: 9.71% ± 1.38%. OKCs: 32.50% ± 3.98%, *p* < 0.001.	OOCs predominantly affect the mandible, exhibit lower proliferative activity than OKCs, and are associated with buccolingual expansion and cortical bone destruction. Due to their lower aggressiveness and recurrence rate, minimally invasive surgical methods like enucleation or decompression followed by enucleation are recommended for treating OOCs.
Orikpete et al. (2020) [125]	To compare the proliferative capacity and antiapoptotic capacity of unicystic ameloblastoma (UA), odontogenic keratocyst (OKC), dentigerous cyst (DC), and radicular cyst (RC) by assessing the Ki-67 labeling index (LI) and Bcl-2 LI, respectively.	Study Type: Retrospective analysis. Sample Size: 23 histopathologically diagnosed UAs, 6 OKCs, 8 DCs, and 10 RCs were selected from archival specimens. Country/Region: Nigeria.	Five micrometer thick sections were made from the tissue blocks and mounted on silanized glass slides. Immunohistochemistry was performed using Ki-67 and Bcl-2 primary antibodies, followed by appropriate detection and staining procedures.	Histopathological examination was used for diagnosis, confirmed by hematoxylin and eosin staining of fresh sections from the tissue blocks.	Ki-67 Expression: UA: 26.1%. OKC: 66.7%; DC: 12.5%; RC: 10.0%. The mean Ki-67 LI was 1.3% for UA, 7.7% for OKC, 1.7% for DC, and 15.3% for RC. Bcl-2 Expression: UA: 69.6%. OKC: 83.3%. DC: 62.5%. RC: 50.0%. The mean Bcl-2 LI was 44.7% for UA, 58.8% for OKC, 5.2% for DC, and 10.3% for RC. Statistical Significance: Significant differences in Ki-67 LI between UA and OKC (*p* = 0.024). Significant differences in Bcl-2 LI between UA and DC (*p* = 0.048), and between OKC and both DC (*p* = 0.026) and RC (*p* = 0.049).	Ki-67 LI: UA: 1.3%. OKC: 7.7%. DC: 1.7%. RC: 15.3%. Significant difference between UA and OKC (*p* = 0.024). Bcl-2 LI: UA: 44.7%. OKC: 58.8%. DC: 5.2%. RC: 10.3%. Significant differences between UA and DC (*p* = 0.048), and between OKC and both DC (*p* = 0.026) and RC (*p* = 0.049).	The Ki-67 LI may help differentiate OKC from UA, and the Bcl-2 LI may be useful in differentiating UA from DC, as well as OKC from DC and RC.
Lafuente-Ibáñez de Mendoza et al. (2022) [126]	The aim of this study is to present and discuss the salient clinicopathological features, differential diagnosis, and epithelial immunohistochemical profile of three additional cases of peripheral odontogenic keratocyst (POKC) and to present a review of the literature.	Study Type: Case series and literature review. Sample Size: 3 new cases of POKC (2 women and 1 man; age range: 14–74 years).	Immunohistochemical study included CK7, CK14, CK19, and Ki-67. A systematic review of the literature was performed using PubMed, Scopus, and Web of Science databases.	Diagnosis of POKC was based on clinicopathological features and immunohistochemical profile.	All cases were in the anterior gingiva (2 in maxilla and 1 in mandible). None of the cases corresponded to Gorlin–Goltz syndrome. High expression of CK14 in all cases, CK19 and CK7 were only focally positive, and Ki-67 expression was in the basal and parabasal cells in all cases.	Not applicable.	POKC is a rare gingival lesion that seems to originate from remnants of dental lamina or from the basal cells of the gingival epithelium and presents a similar histopathology as compared to intraosseous OKC.

### 4.7. Therapeutic Insights and Surgical Management 

Understanding the molecular and biochemical underpinnings of odontogenic lesions, particularly odontogenic keratocysts (OKCs), has significantly improved their therapeutic management and surgical outcomes. Insights into the biochemical behavior of these lesions have led to the refinement of surgical techniques and the development of treatments tailored to their specific pathophysiological features.

Marsupialization, a pre-surgical technique used for OKCs, not only reduces the size of the lesion but also induces biochemical changes within the cyst, such as increased Slug expression [127]. These changes are associated with fibrosis of the cyst wall, which facilitates easier surgical removal and reduces the likelihood of aggressive recurrence. The biochemical insights gained from studying odontogenic lesions have led to significant improvements in surgical management. Techniques like marsupialization, which have been shown to alter biochemical markers within the cyst, are now routinely used to prepare lesions for less invasive surgery, reducing the risk of recurrence. The use of pre-surgical marsupialization based on biochemical marker changes exemplifies how molecular insights are integrated into surgical planning, enhancing therapeutic outcomes by modifying the biological behavior of lesions before more definitive surgical interventions.

These therapeutic insights emphasize the importance of considering the biological behavior of lesions in surgical planning, moving beyond mere removal to positively influencing the lesion’s biochemical environment. Utilizing biochemical markers like Slug in surgical planning allows for more customized and effective interventions, aiming to minimize the risk of recurrence and enhance overall treatment outcomes.

Research into the effects of marsupialization has shown that this procedure significantly increases epithelial thickness and collagen production within the cyst wall [127]. These changes are crucial for reducing the size and aggressiveness of OKCs, facilitating their surgical management. Furthermore, the study by Baris et al. highlighted that marsupialization leads to a significant reduction in the radiographic size of OKCs and an increase in fibrosis, which are key factors in preventing recurrence [127].

The potential for new therapeutic targets based on the molecular and biochemical profiles of odontogenic lesions points towards an era of targeted, specific treatments. For instance, targeting pathways involved in epithelial–mesenchymal transition (EMT) and inflammation could provide new avenues for therapy. The increased expression of Slug post-marsupialization indicates its role in EMT and fibrosis, suggesting that therapies targeting Slug could enhance the efficacy of marsupialization and other surgical interventions.

The advancements in understanding the molecular and biochemical behavior of odontogenic lesions have significant implications for personalized therapy. By integrating molecular insights into surgical planning and postoperative management, clinicians can tailor interventions to the specific characteristics of each lesion, improving patient outcomes. The use of targeted therapies alongside traditional surgical methods could further reduce recurrence rates and enhance the overall effectiveness of treatment.

In summary, the study of the molecular and biochemical aspects of odontogenic lesions, particularly OKCs, has led to significant advancements in their therapeutic management. The integration of biochemical markers into surgical planning and the development of targeted therapies promise to improve patient outcomes by aligning treatment strategies with the underlying causes of lesion behavior [127] (Table 7).

### 4.8. Emerging Markers and Therapeutic Targets 

The treatment landscape for odontogenic lesions, such as odontogenic keratocyst (OKC), adenomatoid odontogenic tumor (AOT), and ameloblastoma (AB), is evolving rapidly due to new discoveries in molecular markers and therapeutic targets like survivin, EGFR, BMP4, FOXN1, and paxillin [128,129,130,131]. These markers are shifting treatment paradigms from traditional surgical interventions to innovative, targeted therapies that address the underlying molecular and genetic drivers of these lesions.

Research into these molecular pathways and genetic mutations has unveiled new therapeutic opportunities. For instance, the roles of molecules such as survivin and EGFR suggest novel approaches for managing lesion growth. Studies have demonstrated that survivin expression is highest in ameloblastoma, followed by OKC, AOT, and reduced enamel epithelium, suggesting that survivin plays a role in inhibiting apoptosis and influencing the biological behavior of these lesions [128]. Similarly, EGFR and survivin have been shown to play crucial roles in the pathogenesis of ameloblastoma, OKC, and calcifying odontogenic cyst, highlighting the potential of targeting these markers in therapeutic approaches [129].

Differences in BMP4 and FOXN1 expression are opening new diagnostic and treatment avenues, potentially allowing for the modulation of cellular behaviors within lesions. Higher expression of BMP4 and FOXN1 in orthokeratinized odontogenic cysts (OOCs) compared to OKCs suggests a higher level of activation of pathways involved in more mature epithelial differentiation in OOCs, potentially contributing to their more benign behavior [130]. This distinction could aid in differential diagnosis and guide targeted therapeutic strategies.

The discovery of new molecular markers and therapeutic targets is transforming the treatment landscape for odontogenic lesions. The identification of EMT-related markers such as Snail and Slug in odontogenic cysts has prompted the exploration of EMT inhibitors as potential therapeutic options, aiming to prevent the invasive progression of these lesions. Significant expression of EMT markers like Snail and Slug in keratocystic odontogenic tumors (KOTs) suggests their role in EMT induction and potential as targets for therapeutic intervention [132].

The exploration of markers related to cell growth, apoptosis, and EMT is leading to therapies that directly target these cellular processes. Such targeted approaches are part of a broader shift towards precision medicine in the treatment of odontogenic lesions, aiming for more effective management with fewer adverse effects and more personalized treatment plans. Differential protein expressions in peripheral ameloblastoma and oral basal cell carcinoma have been shown to aid in accurate classification and tailored treatments [133].

Understanding the molecular and biochemical underpinnings of odontogenic lesions, particularly OKCs, has significantly improved their therapeutic management and surgical outcomes. Insights into the biochemical behavior of these lesions have led to the refinement of surgical techniques and the development of treatments tailored to their specific pathophysiological features. For example, marsupialization, a pre-surgical technique used for OKCs, not only reduces the size of the lesion but also induces biochemical changes within the cyst, such as increased Slug expression [127]. These changes are associated with fibrosis of the cyst wall, which facilitates easier surgical removal and reduces the likelihood of aggressive recurrence. Marsupialization significantly reduces the size of OKCs and increases epithelial thickness and collagenization, suggesting fibrosis and cyst wall strengthening, thus supporting its use as an effective treatment for reducing OKC size and potential recurrence [127].

The potential for new therapeutic targets based on the molecular and biochemical profiles of odontogenic lesions points towards an era of targeted, specific treatments. These advancements promise to align therapeutic strategies more closely with the underlying causes of a lesion’s behavior, enhancing the effectiveness of interventions and leading to better patient outcomes [128,129,130,131].

In summary, the evaluation and incorporation of molecular and biochemical markers in the management of odontogenic lesions represents a significant advancement in the field. These markers provide crucial insights into how these lesions develop and respond to treatments, improving the prediction of outcomes and enabling more effective planning and execution of therapeutic strategies. Ongoing research into these markers is vital for refining management approaches and achieving better patient care outcomes (Table 8).

**Table 8:** Markers and therapeutic targets (OKC, AOT, AB, Survivin, EGFR, BMP4, FOXN1, and paxillin).

Authors	Objective	Study Details	Marker Identification Method	Cyst/Tumor Diagnosis Method	Results	Statistical Estimates	Conclusion
Latha et al. (2023) [128]	To assess the anti-apoptotic survivin expression in reduced enamel epithelium, adenomatoid odontogenic tumor, odontogenic keratocyst, and ameloblastoma.	Study Type: Quantitative analysis. Sample Size: 48 samples (12 each) of reduced enamel epithelium (REE), adenomatoid odontogenic tumor (AOT), odontogenic keratocyst (OKC), and ameloblastoma. Country/Region: India.	Immunohistochemistry was performed using survivin antibody. The sections were stained with hematoxylin and eosin for confirmatory diagnosis before immunohistochemical analysis. The slides were examined under a BX43 microscope with a ProgRes microscope camera, and the survivin expression was analyzed.	Histopathological examination using routine hematoxylin and eosin staining, followed by immunohistochemical analysis with survivin antibody.	Survivin Expression: Total Positive Cells: REE: 313.3; AOT: 1930.16; OKC: 2153.583; ameloblastoma: 2399.5823. Nuclear Expression: REE: 73.91; AOT: 270.83; OKC: 358.66; ameloblastoma: 379.663. Cytoplasmic Expression: REE: 169.833; AOT: 1029.833, OKC: 1003.58; ameloblastoma: 1180.50. Membrane Expression: REE: 20.67; AOT: 89.66; OKC: 174; ameloblastoma: 180.0833. Cytoplasm Membrane Expression: REE: 67.9167; AOT: 453.583; OKC: 617.33; ameloblastoma: 659.416. Intensity of Staining: Mild: REE: 50%; AOT: 25%; OKC: 25%; ameloblastoma: 17%. Moderate: REE: 33%; AOT: 33%; OKC: 25%; ameloblastoma: 42%. Intense: REE: 17%; AOT: 42%; OKC: 50%; ameloblastoma: 50%. Survivin expression was highest in Ameloblastoma, followed by OKC, AOT, and REE. The expression showed significant statistical differences (*p* < 0.05).	Total Positive Cells: *p* = 0.00657. Nuclear Expression: *p* = 0.00219. Cytoplasmic Expression: *p* = 0.00213. Membrane Expression: *p* = 0.000542. Cytoplasm Membrane Expression: *p* = 0.00101. Intensity of Staining: *p* = 0.00005987.	High survivin expression was observed in Ameloblastoma, followed by OKC, AOT, and REE. The expression of survivin in these odontogenic cysts and tumors suggests its role in the inhibition of apoptosis and its potential as a therapeutic target. Higher survivin expression indicates worse prognosis, and its study may aid in understanding the biological behavior of odontogenic cysts and tumors.
Baddireddy et al. (2023) [129]	To assess and compare the expression of EGFR and survivin in ameloblastoma (AB), odontogenic keratocyst (OKC), and calcifying odontogenic cyst (COC).	Study Type: Immunohistochemical study. Sample Size: 30 ABs, 15 OKCs, and 10 COCs. Country/Region: India and USA.	Immunohistochemistry was performed using primary antibodies against EGFR and survivin. The staining procedure included dewaxing, rehydration, antigen retrieval, blocking, primary and secondary antibody incubation, and chromogen development. The slides were examined under an Olympus BX51 research microscope.	Diagnosis was confirmed by examining archival hematoxylin and eosin (H&E) slides.	EGFR Expression: EGFR positivity was found in all cases. Predominant cytoplasmic staining with variations in intensity. Intensity: AB (*p* = 0.007), OKC (*p* = 0.005), COC (*p* = 0.006). IRS Scores: Significant difference between lesions (*p* = 0.02). Survivin Expression: 96% positive in AB, 100% positive in OKC and COC. Predominant cytoplasmic staining with variations in intensity. Intensity: Significant difference in AB peripheral and central cells (*p* = 0.03). IRS Scores: Significant difference between study groups (*p* = 0.001).	EGFR: IRS Scores: AB (*p* = 0.02), OKC (*p* = 0.005), COC (*p* = 0.006). Intensity: AB (*p* = 0.007), OKC (*p* = 0.005), COC (*p* = 0.006). Survivin: IRS Scores: AB (*p* = 0.03), OKC (*p* = 0.09), COC (*p* = 0.06). Intensity: AB (*p* = 0.03), OKC (*p* = 0.09), COC (*p* = 0.06).	The study provides insight into the role of EGFR and survivin in the pathogenesis of AB, OKC, and COC. OKC appears to be more aggressive than AB and COC due to its higher IRS scores. The study highlights the potential for EGFR and survivin as targets for therapeutic interventions in these lesions.
Thermos et al. (2022) [130]	To compare BMP4 and FOXN1 expression in orthokeratinized odontogenic cysts (OOCs) and odontogenic keratocysts (OKCs) to investigate their role in epithelial differentiation.	Study Type: Immunohistochemical comparison. Sample Size: 20 primary sporadic OKCs and 16 OOCs.	Immunohistochemistry was used to assess the expression of BMP4 and FOXN1 in the epithelial and connective tissues of OKC and OOC samples.	The diagnosis was based on histological examination.	BMP4 Expression: Epithelial: Detected in 81.25% OOC vs. 35% OKC. Connective Tissue: Observed in 65% OKC and 75% OOC. FOXN1 Expression: Detected in 75% OOC vs. 30% OKC. BMP4 epithelial and connective tissue positivity and FOXN1 epithelial positivity: 56.25% OOC vs. 10% OKC. Greater expression of BMP4 and FOXN1 in OOC suggests greater activation of this pathway in OOC, contributing to its more mature epithelium and resemblance to an epidermal phenotype.	Not specified.	The greater expression of BMP4 and FOXN1 in OOC suggests a more mature epithelial phenotype and a greater activation of the BMP4/FOXN1 pathway in OOC compared to OKC. This indicates a role in the differing biological behavior and differentiation of these cysts.
Zhang et al. (2018) [131]	To explore the potential involvement of Fra-1, c-Jun, and c-Fos, three vital members of the AP-1 complex, in the pathogenesis of odontogenic keratocysts (OKCs).	Study Type: Immunohistochemical and RT-qPCR analysis. Sample Size: 10 normal oral mucosa (OM), 10 dentigerous cysts (DC), and 32 OKC specimens.	Immunohistochemistry and real-time-quantitative polymerase chain reaction (RT-qPCR) were used to investigate the expression levels of Fra-1, c-Jun, and c-Fos. Double-labelling immunofluorescence analysis was also used to confirm the associations.	Diagnosis was based on histological examination and immunohistochemical analysis.	Fra-1, c-Jun, and c-Fos Expression: Increased significantly in OKCs compared to OM and DC tissue samples. Positively associated with the expression levels of Ki-67, PCNA, and Bcl-2. Analysis Methods: Double-labelling immunofluorescence analysis Hierarchical analysis.	Not specified.	This study revealed for the first time that Fra-1, c-Jun, and c-Fos were overexpressed in OKCs and had a close correlation with proliferation and anti-apoptosis potential of OKCs.
Pinheiro et al. (2020) [134]	To evaluate tryptase and E-cadherin protein expression in odontogenic keratocysts (OKCs) and radicular cysts (RCs) and their relationship with lesion size.	Study Type: Immunohistochemical analysis. Sample Size: 30 OKCs and 30 RCs. Country/Region: Brazil.	Immunohistochemistry was used to assess the expression of tryptase and E-cadherin in tissue samples. Tryptase expression was quantitatively assessed by counting mast cells, and E-cadherin expression was semi-quantitatively analyzed by estimating the proportion of positive cells.	The diagnosis was based on histological examination and radiographic measurements of the cystic lesion sizes.	Tryptase Expression: Higher mast cell means were found in RCs compared to OKCs. Degranulated mast cells were predominant in both OKCs and RCs. Negative correlation between E-cadherin expression and total number of mast cells, degranulated mast cells, and lesion size. E-cadherin Expression: Negative correlation with total number of mast cells (*p* = 0.011), degranulated mast cells (*p* = 0.040), and degranulated mast cells in both superficial (*p* = 0.035) and deep connective tissues (*p* = 0.009). Lesion Size: RCs: 67% ≤ 2 cm, 27% > 2 to 4 cm, 6% > 4 cm. OKCs: 47% ≤ 2 cm, 37% > 2 to 4 cm, 16% > 4 cm. Negative correlation between lesion size and total number of mast cells in the epithelium (*p* = 0.016) and degranulated mast cells in the epithelium (*p* = 0.049).	*p*-values: <Total number of mast cells in epithelium: *p* = 0.016. Degranulated mast cells in epithelium: *p* = 0.049. E-cadherin expression and total number of mast cells: *p* = 0.011. E-cadherin expression and degranulated mast cells: *p* = 0.040. E-cadherin expression and degranulated mast cells in superficial connective tissue: *p* = 0.035. E-cadherin expression and degranulated mast cells in deep connective tissue: *p* = 0.009	The higher expression of tryptase in degranulated mast cells is linked to lower expression of E-cadherin, suggesting a change in epithelial permeability and contributing to increased cystic content and lesion growth. Mast cells in RCs may initiate cystic formation, while in OKCs, they act in more advanced stages, contributing to bone resorption and lesion expansion.
Porto et al. (2016) [132]	To evaluate the epithelial–mesenchymal transition (EMT) in keratocystic odontogenic tumors (KOTs) by assessing the immunoexpression of E-cadherin, N-cadherin, Snail, and Slug and comparing them to radicular cysts and dental follicles.	Study Type: Immunohistochemical study. Sample Size: 32 KOTs, 15 radicular cysts, and 8 dental follicles.	Immunohistochemistry was used to evaluate the expression levels of E-cadherin, N-cadherin, Snail, and Slug in tissue samples.	Diagnosis was based on histological examination and immunohistochemical analysis of the collected samples.	E-cadherin: Preserved in most cases of KOT. N-cadherin: Increased in the tumor epithelium, positively correlated with heterogeneous and nuclear immunoexpression of Slug in the epithelium. Also correlated with high Snail immunoexpression. Slug: Heterogeneous and nuclear expression in the epithelium, correlated with high Snail expression. Snail: High immunoexpression in KOTs. Stroma: N-cadherin positively correlated with Slug.	Not specified.	The high immunoexpression of Snail and nuclear Slug in KOTs suggests these proteins act as transcription factors without necessarily participating in “cadherin switching”. However, the understanding of their role in inducing EMT in odontogenic tumors is still limited.
Singh et al. (2023) [13]	To analyze the immunohistochemical expression of paxillin in ameloblastoma (AB) and odontogenic keratocyst (OKC) to appraise their roles in cell-matrix interactions.	Study Type: Observational study. Sample Size: 60 cases (30 AB and 30 OKC). Age Range: OKC: 10–20 years: 2 cases. 21–30 years: 16 cases. 31–40 years: 6 cases. 41–50 years: 3 cases. 50 years: 3 cases. AB: 10–20 years: 6 cases. 21–30 years: 11 cases. 31–40 years: 5 cases. 41–50 years: 5 cases. 50 years: 3 cases. Country/Region: India.	Immunohistochemistry was used to stain tissue sections with a paxillin antibody. Staining intensity and quantitative staining were evaluated and scored.	Diagnosis was confirmed using histopathological criteria and hematoxylin and eosin staining.	Staining Intensity: Score 0 (No staining): OKC 1 (3%), AB 0 (0%). Score 1 (Weak staining): OKC 13 (43%), AB 6 (20%). Score 2 (Moderate staining): OKC 10 (33%), AB 6 (20%). Score 3 (Strong staining): OKC 5 (17%), AB 8 (27%). Score 4 (Very strong staining): OKC 1 (3%), AB 10 (33%). Statistical Comparison: Significant (*p* = 0.013). Quantitative Staining: Score 0 (No staining): OKC 1 (3%), AB 0 (0%). Score 1 (<25% of cells): OKC 9 (30%), AB 5 (17%). Score 2 (25–50% of cells): OKC 5 (17%), AB 5 (17%). Score 3 (50–75% of cells): OKC 5 (17%), AB 10 (33%). Score 4 (>75% of cells): OKC 10 (33%), AB 10 (33%). Statistical Comparison: Non-significant (*p* = 0.432). Final Summation Score: Score 0 (No staining): OKC 1 (3%), AB 0 (0%). Score 1–4 (Weak staining): OKC 16 (53%), AB 11 (37%). Score 5–8 (Strong staining): OKC 13 (43%), AB 19 (63%). Statistical Comparison: Non-significant (*p* = 0.503). Gender-wise Comparison: OKC: Significant (*p* < 0.05). AB: Quantitative staining and final summation significant (*p* < 0.001 and *p* = 0.027), staining intensity non-significant (*p* = 0.091). Age-wise Comparison: OKC: Weak staining predominant in 21–30 years (53%). AB: Strong staining predominant in 21–30 years (66%)	*p*-values: Staining Intensity: *p* = 0.013. Quantitative Staining: *p* = 0.432. Final Summation Score: *p* = 0.503. Gender-wise Comparison in OKC: *p* < 0.05. Gender-wise Comparison in AB: Quantitative staining *p* < 0.001, Final summation *p* = 0.027, Staining intensity *p* = 0.091	Paxillin expression is significant in the epithelial lining of both OKC and AB, suggesting its role in cell-matrix interactions and tumorigenesis. The expression pattern indicates its involvement in the biological behavior of these odontogenic lesions. Further studies with larger sample sizes and molecular analyses are needed to confirm paxillin’s exact role and potential as a therapeutic target.
Cesinaro et al. (2020) [135]	To assess the expression of calretinin in odontogenic keratocysts (OKCs) and basal cell carcinomas (BCCs) in sporadic and Gorlin–Goltz syndrome (GGS) cases.	Study Type: Immunohistochemical analysis. Sample Size: 28 OKCs: 16 sporadic OKCs from 15 patients, 12 GGS-related OKCs from 11 patients. 34 BCCs: 19 BCCs and 2 cutaneous keratocysts from 4 GGS patients, 15 sporadic BCCs and 3 steatocystomas. Age Range: Sporadic OKCs: 10 to 61 years (Mean: 39.6 years, SD: 14.71) GGS-OKCs: 26 to 44 years (Mean: 32.7 years, SD: 5.82) Country/Region: Italy.	Immunohistochemistry was performed on 4-μm thick sections using anti-calretinin SP65 pre-diluted monoclonal rabbit antibody. Immunostaining was evaluated as negative, focally positive (<5% of cells), or positive (>5% of cells).	Diagnosis was based on histological examination according to the WHO classification of head and neck tumors.	Calretinin Expression in OKCs: GGS-OKCs: 10 negative, 2 focally positive. Sporadic OKCs: 6 negative, 6 focally positive, 4 diffusely positive. Significant difference between sporadic and GGS-OKCs (*p* = 0.02). Calretinin Expression in BCCs: GGS-BCCs: 14 negative, 4 focally positive, 1 diffusely positive. Sporadic BCCs: 7 negative, 8 focally positive. No significant difference between GGS and sporadic BCCs. Calretinin Expression in Cutaneous Cysts: GGS–cutaneous keratocysts: 2 negatives. Sporadic Steatocystomas: 1 negative, 1 focally positive, 1 diffusely positive	*p*-values: Calretinin expression in OKCs: *p* = 0.02. Calretinin expression in BCCs: Not significant. Calretinin expression in cutaneous cysts: Not significant.	Calretinin expression is significantly lower in GGS-OKCs compared to sporadic OKCs, suggesting a potential link between SHH pathway dysfunction and calretinin expression in GGS-related tumors. However, calretinin’s value in differential diagnosis between sporadic and syndromic tumors appears limited.
Galvão et al. (2013) [136]	To perform an immunohistochemical assessment of protein 53 (p53), proliferating cell nuclear antigen (PCNA), B-cell lymphoma 2 (bcl-2), and murine double minute 2 (MDM2) expression in odontogenic cysts and keratocystic odontogenic tumor (KCOT), analyzing their correlation with the biological behavior of these lesions.	Study Type: Immunohistochemical analysis. Sample Size: 11 radicular cysts, 11 dentigerous cysts, 11 KCOTs.	The streptavidin-biotin-peroxidase method was used with antibodies against p53, PCNA, bcl-2, and MDM2 proteins.	Diagnosis was based on histological examination and immunohistochemical analysis.	PCNA: Immunopositivity observed in all cases, predominantly in the suprabasal layer of KCOT epithelial lining (SD ± 19.44), but no significant differences among the groups. Bcl-2: Immunoexpression observed especially in the basal layer of KCOT. PCNA LI: Significantly higher than bcl-2 LI in KCOT. MDM2 and p53: Immunoexpression not detected in the lesions studied. KCOT: Showed different immunoexpression of proliferation and apoptosis markers compared to other odontogenic cysts.	Non-parametric Mann–Whitney U-test and Kruskal–Wallis test (*p* ≤ 0.05).	The results suggest that KCOT presents distinct biological behavior compared to odontogenic cysts, in terms of proliferation, apoptosis, and differentiation. This supports the neoplastic nature of KCOT.
Tenório et al. (2018) [11]	To investigate the expression of Bcl-2, Bax, and p53 to better understand the possible role of these proteins in ameloblastomas (AMBs), odontogenic keratocysts (OKCs), and adenomatoid odontogenic tumors (AOTs).	Study Type: Immunohistochemical analysis. Sample Size: 20 AMBs, 20 OKCs, 20 AOTs.	Immunohistochemistry technique was performed for the antibodies p53, Bcl-2, and Bax. Immunoreactivity was observed in the epithelial component.	Diagnosis was based on histological examination and immunohistochemical analysis.	Expression of Proteins: All lesions exhibited staining for p53, Bcl-2, and Bax. Statistical Analysis: No statistically significant associations between the expression of proteins and the lesions. Correlations: Positive correlation between the expression of p53 and Bcl-2 (r = 0.200). Negative correlation between p53 and Bax expressions (r = −0.100). p53 and Bax were similarly expressed between AMBs and OKCs. Bcl-2 was similarly expressed in AMBs and AOTs.	Kruskal–Wallis and Spearman tests (*p* < 0.05). Correlation Coefficients: p53 and Bcl-2: r = 0.200. p53 and Bax: r = −0.100	Apoptosis regulatory proteins, as well as cell cycle proteins, are differently expressed in epithelial odontogenic lesions. Their expression is possibly related to the biological behavior of AMBs, OKCs, and AOTs.
Ghafouri-Fard et al. (2021) [137]	To summarize the current data on the expression patterns of genes in ameloblastoma (AB), dentigerous cyst (DC), and odontogenic keratocyst (OKC), and to examine the association between genetic polymorphisms and the development of these lesions.	Study Type: Review. Sample Size: Various studies and samples were mentioned within the review. Country/Region: Iran.	Gene expression profiling, immunohistochemistry, cDNA microarray, RT-qPCR, Western blotting, loss of heterozygosity (LOH), PCR-RFLP, next-generation sequencing, methylation-specific PCR, and microarray studies.	Diagnosis was based on histological examination and various genetic and molecular assays.	Ameloblastoma (AB): Dysregulation of genes such as FOS, TNFRSF1A, SHH, TRAF3, ARHGAP4, DCC, CDH12 and 13, TDGF1, TGFB1, WNT1, IGF2, P63, WT1, IL-6, PTEN, COX-2, and many others. High incidence of BRAF V600E and SMO L412F mutations. Overexpression of long non-coding RNAs (lncRNAs) such as ENST00000512916 and KIAA0125. Genetic polymorphisms in genes like MMP9, APC, XRCC1, P53, RECK, and PTCH1. Dentigerous Cysts (DCs): Differential expression of genes related to extracellular matrix formation, adhesion, invasion, metabolic pathways, cell signaling, cytokine functions, inflammation, and immune responses. Genetic polymorphisms are associated with the PTCH gene region. Odontogenic Keratocysts (OKCs): Overexpression of genes like PTCH, SHH, SMO, GLI1, CCND1, and BCL2. Genetic polymorphisms and mutations in PTCH1, P53, IL-1, survivin gene promoter, and MIR15A/MIR16-1. Lower expression of calretinin in syndromic OKCs compared to sporadic cases.	Not specified.	The review highlights the significant role of dysregulated genes, genetic polymorphisms, and miRNA/lncRNA expressions in the pathogenesis of AB, DC, and OKC. These molecular markers can potentially aid in the diagnosis, prognosis, and development of therapeutic approaches for these odontogenic lesions.
Kim et al. (2014) [133]	To compare the protein expression profiles of peripheral ameloblastoma (PA) and oral basal cell carcinoma (OBCC) occurring in the same mandibular molar area to better understand their tumorigenesis.	Study Type: Case study with immunohistochemical examination. Sample Size: One case of PA in a 61-year-old male and one case of OBCC in a 33-year-old male. Country/Region: Korea.	Immunohistochemistry using 50 antisera selected for important signaling pathways. Staining was evaluated and confirmed through repeated testing.	Histological examination and immunohistochemical analysis based on tissue samples.	PA: Strong positive for ameloblastin, KL1, p63, carcinoembryonic antigen (CEA), focal adhesion kinase (FAK), and cathepsin K. Slightly positive for amelogenin, Krox-25, E-cadherin, and PTCH Exhibited odontogenic differentiation and active ectomesenchymal interaction. Higher positivity for proteins associated with odontogenic epithelium, epithelial adhesion, and bone resorption. OBCC: Strong positive for EpCam, MMP-1, α1-antitrypsin, CK-7, p53, survivin, pAKT1, TGF-β1, N-RAS, TGase-1, and TNFα. Consistently positive for β-catenin, MMP-2, cathepsin G, TGase-2, SOS-1, SHH, and β-defensins 1, 2, and 3 exhibited basaloid epidermal differentiation influenced by growth factor/cytokine-related signals. Higher positivity for proteins associated with proliferation, apoptosis, and inflammation. Common Markers: Both PA and OBCC showed positive reactions for PCNA, NFkB, MMP-9, eIF5A, BCL-2, PARP, PIM1, NF-1, HSP-70, 14-3-3, HIF, vWF, and VEGF, indicating similar tumor growth potential.	Not specified.	PA and OBCC differ in their protein expression profiles, with PA showing odontogenic differentiation and OBCC exhibiting basaloid epidermal differentiation. These differences suggest distinct tumorigenesis pathways and could aid in differential diagnosis. Further investigations are required to fully elucidate characteristic protein expressions in these tumors.

## 5. Conclusions

This systematic review has comprehensively explored the roles of immunohistochemical markers in dentigerous cysts (DCs) and odontogenic keratocysts (OKCs) associated with impacted third molars. By synthesizing data from 138 articles, this review highlights the diagnostic, prognostic, and therapeutic importance of markers such as Ki-67, p53, Bcl-2, and PCNA. These markers have proven instrumental in predicting aggressive behavior and guiding management strategies for OKCs, which are prone to aggressive growth and recurrence.

The findings indicate that the elevated expressions of Ki-67 and p53 in OKCs are particularly significant, suggesting that these markers can critically inform clinical decisions regarding the timing and extent of surgical interventions. Additionally, the identification of PTCH1 gene mutations and alterations in the SHH pathway presents promising targets for developing novel therapeutic approaches, potentially leading to more effective treatments tailored to the genetic profiles of individual lesions.

However, this review acknowledges several limitations, including the heterogeneity of the study designs, sample sizes, and methodologies used, which may affect the generalizability of the findings. Most studies were limited by small sample sizes and the retrospective nature of data collection, which can introduce bias and limit the applicability of the results to a broader population. Furthermore, the predominance of research from high-resource settings may not accurately represent the global burden and characteristics of these conditions.

To address these limitations, future research should focus on conducting large-scale, multicentric prospective studies that include diverse populations to enhance the external validity of the findings. There is also a pressing need for longitudinal studies to assess the long-term outcomes of different therapeutic interventions and their impact on the patient’s quality of life. Exploring the molecular mechanisms driving the expression of these immunohistochemical markers could uncover additional therapeutic targets. Furthermore, the development of non-invasive diagnostic tools based on these markers could revolutionize the early detection and management of DCs and OKCs, offering substantial improvements in patient care.

In summary, while this review makes significant strides toward understanding the complex pathology of odontogenic cysts and tumors, it also underscores the crucial need for continued research and innovation in this field. Ensuring that future studies address the identified limitations will be essential for producing findings that are robust, replicable, and applicable to diverse patient populations. By integrating immunohistochemical data into clinical practice, clinicians can optimize therapeutic outcomes and reduce the recurrence rates of these potentially aggressive conditions, ultimately advancing patient care in oral and maxillofacial surgery.

## Figures and Tables

**Table 7:** Therapeutic and surgical management (OKCs).

Authors	Objective	Study Details	Marker Identification Method	Cyst/Tumor Diagnosis Method	Results	Statistical Estimates	Conclusion
Baris et al. (2022) [127]	To assess the effect of marsupialization on histomorphological and biochemical markers of odontogenic keratocysts (OKCs).	Study Type: Retrospective analysis. Sample Size: 48 paraffin blocks of 24 OKC cases between 2012 and 2018. Country/Region: Turkey.	Immunohistochemical staining with E-cadherin, Ki67, IL1α, TNFα, Slug, and Snail was performed on 4 µm thick sections of formalin-fixed paraffin OKC sections. The BOND Polymer Refine Detection Kit and BOND Polymer Refine Red Detection Kit were used for staining on the Leica BOND-MAX fully automated IHC and ISH staining system.	Diagnosis was based on histological and histomorphometric analysis, and radiological data including measurements on orthopantomographs.	The majority (70.8%) of OKC cases were in the mandibular posterior region. Marsupialization Period: Mean period was 8.8 ± 6.5 months (range: 3–25 months). Radiographic Findings: Mean size of OKC significantly reduced from 57.1 ± 53.5 mm to 22.6 ± 19.9 mm after marsupialization (*p* = 0.002). Histological Findings: Increased epithelial thickness (*p* = 0.002) and collagen production (*p* = 0.034) post-marsupialization. Positive correlation of inflammation score with TNFα (r: 0.69, *p* < 0.001) and IL-1α (r: 0.58, *p* = 0.008) expressions in connective tissue. Significant increase in Slug expression after marsupialization (*p* = 0.019).	Epithelial Thickness: Increased from 83.46 ± 45.05 µm to 167.39 ± 110.08 µm (*p* = 0.002). Collagen Production: Increased significantly post-marsupialization (*p* = 0.034). Inflammation Scores: Positive correlations with TNFα and IL-1α expressions (*p* < 0.001 and *p* = 0.008, respectively). Slug Expression: Significantly higher in the connective tissue post-marsupialization (*p* = 0.019).	Marsupialization can lead to significant changes in the histomorphology of OKCs, including increased epithelial thickness and collagen production. The increase in Slug expression may contribute to fibrosis, potentially aiding in subsequent surgical procedures.

## Data Availability

Data can be found on PubMed.
